# Localisation of the Putative Magnetoreceptive Protein Cryptochrome 1b in the Retinae of Migratory Birds and Homing Pigeons

**DOI:** 10.1371/journal.pone.0147819

**Published:** 2016-03-08

**Authors:** Petra Bolte, Florian Bleibaum, Angelika Einwich, Anja Günther, Miriam Liedvogel, Dominik Heyers, Anne Depping, Lars Wöhlbrand, Ralf Rabus, Ulrike Janssen‐Bienhold, Henrik Mouritsen

**Affiliations:** 1 Institute for Biology and Environmental Sciences, University of Oldenburg, Oldenburg, Germany; 2 Research Centre for Neurosensory Sciences, University of Oldenburg, Oldenburg, Germany; 3 Max-Planck-Institute for Evolutionary Biology, Plön, Germany; 4 Institute for Chemistry and Biology of the Marine Environment, Carl-von-Ossietzky University, Oldenburg, Germany; 5 Department of Neurobiology, University of Oldenburg, Oldenburg, Germany; Lund University, SWEDEN

## Abstract

Cryptochromes are ubiquitously expressed in various animal tissues including the retina. Some cryptochromes are involved in regulating circadian activity. Cryptochrome proteins have also been suggested to mediate the primary mechanism in light-dependent magnetic compass orientation in birds. Cryptochrome 1b (Cry1b) exhibits a unique carboxy terminus exclusively found in birds so far, which might be indicative for a specialised function. Cryptochrome 1a (Cry1a) is so far the only cryptochrome protein that has been localised to specific cell types within the retina of migratory birds. Here we show that Cry1b, an alternative splice variant of Cry1a, is also expressed in the retina of migratory birds, but it is primarily located in other cell types than Cry1a. This could suggest different functions for the two splice products. Using diagnostic bird-specific antibodies (that allow for a precise discrimination between both proteins), we show that Cry1b protein is found in the retinae of migratory European robins (*Erithacus rubecula*), migratory Northern Wheatears (*Oenanthe oenanthe*) and pigeons (*Columba livia*). In all three species, retinal Cry1b is localised in cell types which have been discussed as potentially well suited locations for magnetoreception: Cry1b is observed in the cytosol of ganglion cells, displaced ganglion cells, and in photoreceptor inner segments. The cytosolic rather than nucleic location of Cry1b in the retina reported here speaks against a circadian clock regulatory function of Cry1b and it allows for the possible involvement of Cry1b in a radical-pair-based magnetoreception mechanism.

## Introduction

Migratory birds use the Earth's magnetic field for orientation and navigation on their migratory journeys [1–3]. However, the molecular mechanisms underlying birds’ magnetic perception are still unknown. It is almost certain that migratory birds use more than one mechanism to detect magnetic information (for review see [4] and [5]). One magnetoreception mechanism might be associated with the ophthalmic branch of the trigeminal nerve [6–9, but see 10] and/or the inner ear lagena [11,12]. Furthermore, strong experimental data support the hypothesis that the magnetic compass is based on a light-dependent, radical-pair-based reaction mechanism [13–17] in both eyes [18–21], and that this information is processed in the thalamofugal visual pathway [22–25]. According to this hypothesis, light absorption by a primary magnetosensitive molecule leads to the formation of an intermediate radical-pair, which can be either in a singlet or triplet excited state, depending on the molecule’s orientation within the ambient magnetic field [13,15,26–33]. If we assume that the state of the transient radical alters the relative photosensitivity of the receptor molecule, radical-pair-mediated magnetoreception would thus allow the detection of the symmetry plane or axial orientation of the field lines, but not their polarity. This is in line with the fact that night-migratory songbirds use an “inclination compass”, which is sensitive to the axis of the Earth’s magnetic field lines, but not to their polarity [1,2].

The identity of any magnetosensitive molecule in the birds’ eye has not yet been confirmed. Migratory birds are oriented in their natural migratory direction under blue and green light but tend to be disoriented under yellow and red light [2,34,35]. These results might suggest that sensory proteins, which form the radical-pairs upon photoexcitation should be excitable by light in the blue-green range. To date, the only known class of proteins found in the bird retina which form radical-pairs upon photoexcitation, are the cryptochromes [15,28–33,36,37] and they absorb in the blue wavelength range [13,36,38,30]. Cryptochromes are closely related to photolyases, a class of flavoproteins that is involved in the repair of UV-light-damaged DNA via electron transfer mechanisms and form radical-pairs when excited by light [38–42]. Cryptochromes do not possess photolyase activity but do form radical-pairs upon photoexcitation [15,28–31,36,37] and they are involved in various blue-light-dependent pathways in several plants and animals (for a general overview see [43–45]). Moreover, cryptochromes of migratory garden warbler (*Sylvia borin*) have been shown to be excited by blue light and produce radical intermediates with millisecond lifetimes, long enough to allow for Earth strengths magnetic field to modulate singlet-triplet interconversion [36]. To date, four different members of the cryptochrome multigene family have been identified in the retina of migratory birds: Cry1a [46–49]; Cry1b [47,49]; Cryptochrome 2 (Cry2) [46,47] and Cryptochrome 4 (Cry4) [50].

In the mammalian retina, cryptochromes are involved in circadian photoentrainment [44,45,51–55]. But are all cryptochromes in the retina of migratory birds also core components of the circadian pacemakers, or is it possible that one (or more) members of this multigene family may act as a magnetic sensor? The sub-cellular localisation of cryptochromes can give important hints to cryptochromes’ functions within the avian retina. Since cryptochromes that are mainly involved in regulating gene expression of the internal circadian clock are predominantly localised in the nucleus [53,56], a cytoplasmic localisation of a cryptochrome type in the bird’s retina could be indicative for a non-clock function. Here we aim to characterise the cellular location of Cry1b in the retina of migratory birds and homing pigeons.

## Results

Cry1a and Cry1b are alternative splice products and differ in their C-terminal region. To document the distribution of Cry1b within the retina and to allow for a comparison of Cry1a and Cry1b localisation, we generated a polyclonal antibody specific for a 17 amino acids peptide at the diagnostic C-terminal end of the garden warbler Cry1b (gwGry1b) protein. Specificity of the affinity-purified antibody was tested in controls by replacing the primary antibody with pre-immune serum, antigen pre-adsorption test, immunoblotting, and using mouse neuroblastoma (N2a) cells recombinantly expressing garden warbler Cry1b and Cry1a proteins.

**Fig 1:**
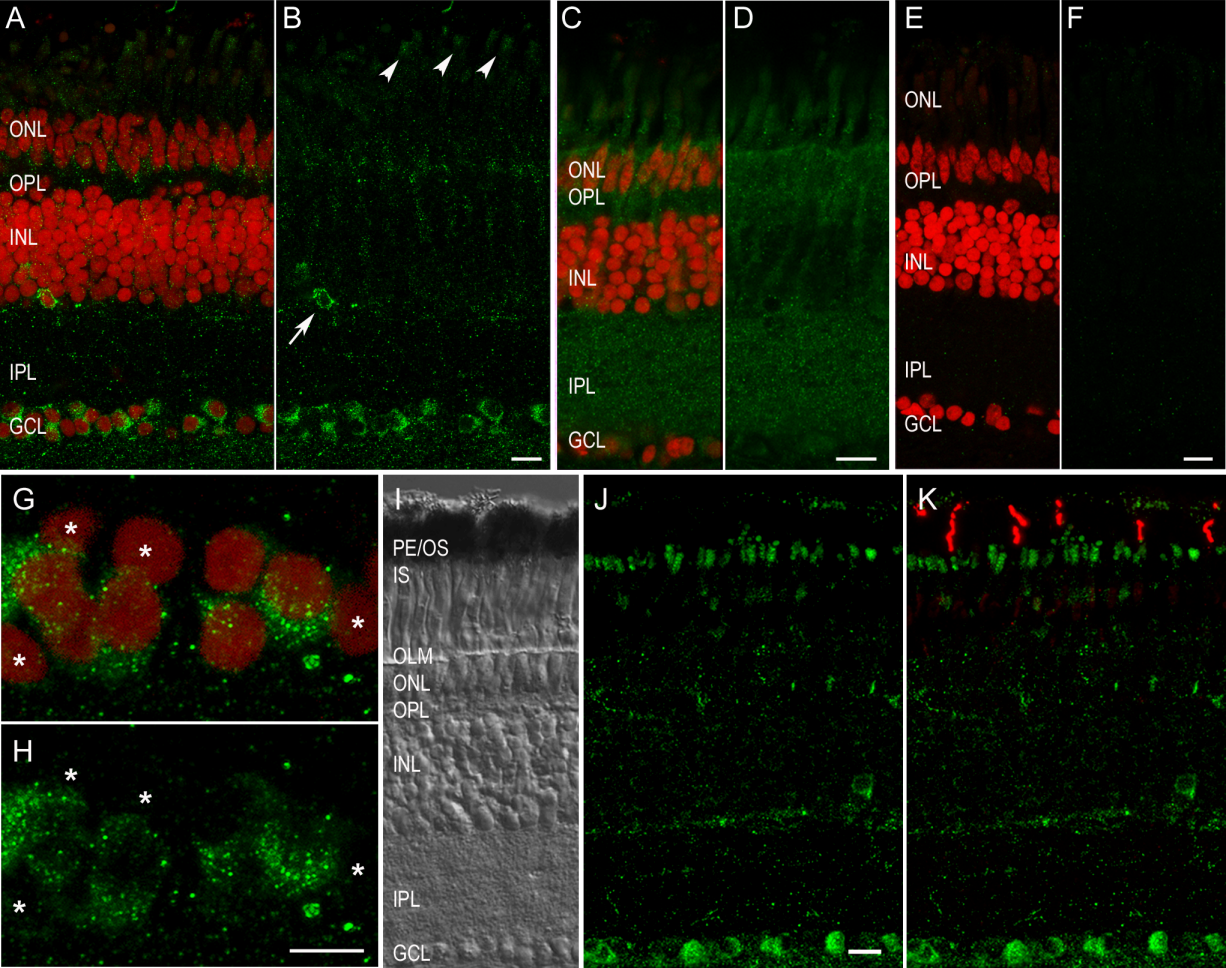
In the pigeon retina, Cry1b was expressed in ganglion cells, putative displaced ganglion cells and photoreceptor inner segments. Vertical slices of pigeon retina labelled with gwCry1b antibody (A, B green) and Roti® Mount nuclear marker (A, red) showed strong expression of Cry1b protein in the cytoplasm of ganglion cells and single cells in the proximal INL (B, arrow) and weak Cry1b expression in the outer parts of photoreceptors (B, arrowheads). This labelling was absent in controls with pre-immune serum (C, D) and in controls with gwCry1b antibody blocked by the accordant gwCry1b peptides (E, F). Projection (10.1 μm) of gwCry1b labelling (G, H green) together with the Roti® Mount nuclear marker (G red) in the ganglion cell layer showed strong Cry1b expression in the cytoplasm of several ganglion cells, whereas weak or no staining was observed in other ganglion cells (G, H asterisks). With the bright field image for comparison (I), Cry1b immunoreactivity (J, K green) in the photoreceptors was located distal to the outer limiting membrane and proximal to the pigment epithelium. Double immunostaining (K) revealed that Cry1b immunoreactivity (K green) in the outer parts of photoreceptors was not present in outer segments of OPN1SW labelled cones (K red), but in the photoreceptor inner segments proximal to the outer segments. Images (A-F) are maximum projections of confocal stacks and were taken from the same experiment with identical microscope settings and without any image adjustments**.** Scale bars: A-F, 10 μm G, H, 5 μm; I, J, K 10 μm. PE, pigment epithelium; OS, photoreceptor outer segments; IS, photoreceptor inner segments; OLM, outer limiting membrane; ONL, outer nuclear layer; OPL, outer plexiform layer; INL, inner nuclear layer; IPL, inner plexiform layer; GCL, ganglion cell layer.

In cryo-sections of pigeon retinae (N = 3), the gwCry1b antibody indicated immunoreactivity in the ganglion cells and single cells in the proximal inner nuclear layer (INL). Moderate intensity immunosignal was also observed in the inner segments of the photoreceptors (Fig 1A and 1B, shown in green). Analysis of gwCry1b immunoreactivity together with a nuclear marker (Fig 1A, shown in red) indicated a cytoplasmic localisation in ganglion and displaced ganglion cells. In addition to the labellings of birds killed after sunset, we also did immunocytochemical stainings of European robins killed around noon, in the afternoon and at 04:00 am in the night. All these samples showed a cytoplasmic labelling of Cry1b in the ganglion cells. The labelled cells in the INL were usually not distinguishable in size from bipolar cells (as seen in Fig 1A), but their scarcity (approximately 5–12 cells per retinal slice) and location in the outermost proximal part of the INL suggested that they were displaced ganglion cells. A more detailed analysis of gwCry1b immunoreactivity in the ganglion cell layer together with a nuclear marker (red) indicated that the amount of Cry1b protein differed between individual GCs (Fig 1G and 1H). While some ganglion cells showed strong immunoreactivity to gwCry1b, others showed little or no staining (Fig 1G and 1H). Double labelling of the gwCry1b antibody with an antibody raised against UV opsin (OPN1SW) indicated that gwCry1b was not present in the outer segments of photoreceptors, as has been described for Cry1a [48], but in the inner segments (Fig 1J and 1K). Both the diffuse background labelling found in all retinal layers and the lack of specific labelling of individual cell populations in control stainings, where we used the pre-immune serum instead of the primary gwCry1b antibody, allowed us to exclude the possibility that the observed Cry1b labelling was due to unspecific reactions with antibodies present before immunisation of the animals (Fig 1C and 1D, shown in green). No Cry1b staining was detected in control stainings, where the gwCry1b antibody was pre-adsorbed (blocked) by gwCry1b peptides. This indicates that the gwCry1b antibody allows for specific detection of epitopes present on the peptides used for immunisation (Fig 1E and 1F).

**Fig 2:**
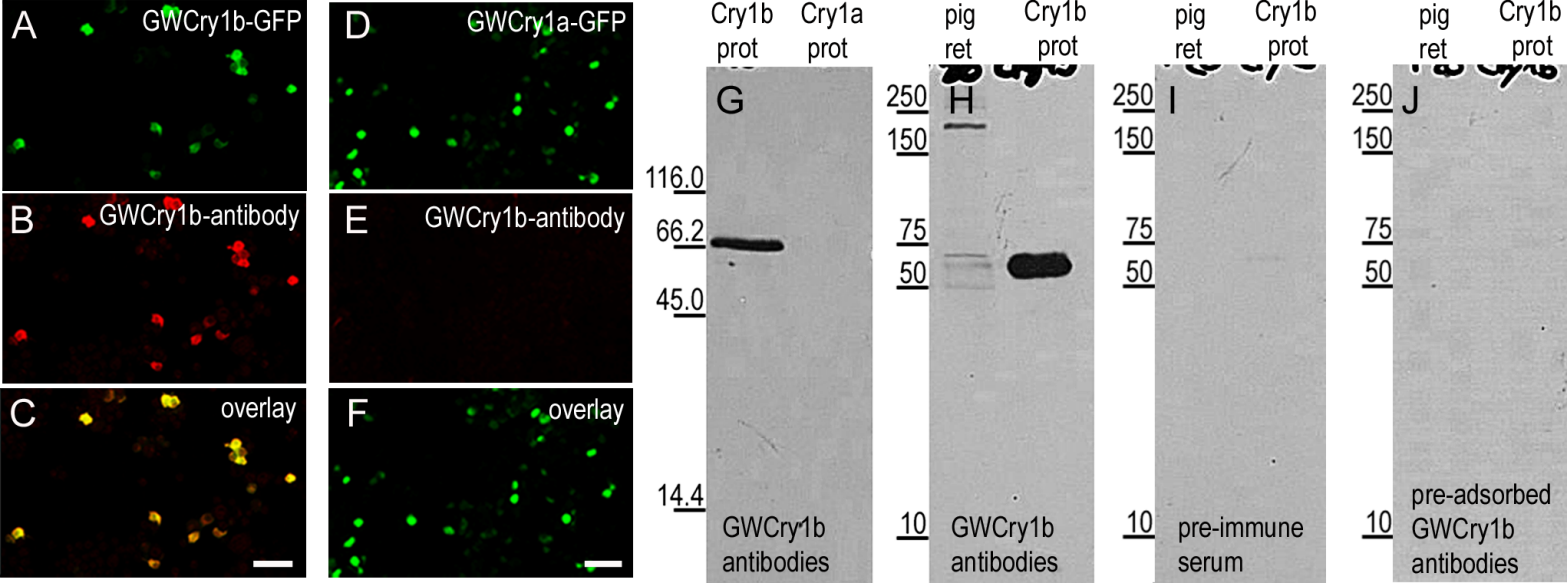
Specificity of gwCry1b antibody. N2a cells expressing a gwCry1b-GFP fusion protein (A green) stained with gwCry1b antibody (B red) indicated that the antibody detects gwCry1b-GFP protein (C yellow). The same labelling of gwCry1a-GFP expressing cells (D-F) indicated no detection of gwCry1a protein. In Western blots, gwCry1b antibody detected a single protein of the expected size (~64 kDa) in samples of purified gwCry1b protein (G left lane) but not in samples of gwCry1a protein (G right lane). Western blots incubated with gwCry1b antibody showed a band of ~64 kDa on retinal total homogenates from pigeons (H left lane) and purified gwCry1b protein (H right lane). No bands were detected in blots incubated with pre-immune serum (I) and gwCry1b antibody blocked by gwCry1b peptides (J). In G-J, the molecular mass in kDa is indicated on the left. All images (A-E) are maximum projections of confocal stacks and were taken from the same experiment with identical microscope settings and without any image adjustments**. **

To confirm that our antibody detects gwCry1b protein, we generated N2a cells recombinantly expressing a protein containing the coding region for retinal gwCry1b fused to green fluorescent protein (GFP). Colocalisation of green fluorescent N2a cells expressing gwCry1b-GFP fusion protein and red-stained gwCry1b antibody indicates that our generated antibody specifically detects gwCry1b protein (Fig 2A–2C). The same procedure with gwCry1a-GFP expressing cells indicates the absence of cross-reactivity of gwCry1b antibody with gwCry1a protein and GFP protein (Fig 2D–2F). We omitted the gwCry1b antibody or replaced it by pre-immune serum to show that there is no cross-reaction of gwCry1a-GFP expressing cells with the secondary antibody or with antibodies present before immunisation of the rabbits (see S1 Fig). The specificity of gwCry1b antibody was also tested using Western blot analysis. In Western blot analyses, the gwCry1b antibody detected a single protein of the expected size (~64 kDa) in samples of purified gwCry1b protein (Fig 2G). Furthermore, mass spectrometric analysis of the respective SDS-PAGE band confirmed the presence of Cry1b (see S1 and S2 Tables). No band was labelled in samples of purified gwCry1a protein (Fig 2G). The appropriate band of ~64 kDa, indicative for Cry1b, was also detected in retinal lysates from pigeons (Fig 2H). In the retinal lysates, there were three additional bands. Such extra bands are often seen in Western blots and might arise from gwCry1b protein oligomerisation, phosphorylation or dephosphorylation, which alter the molecular weight of the gwCry1b protein. No antibody labelling was detected in negative controls after stripping and re-probing the blot with pre-immune serum (Fig 2I) and gwCry1b antibody pre-adsorbed with the appropriate peptides (Fig 2J).

The cellular localisation of Cry1b within the retina was confirmed by an additional polyclonal antibody (erCry1b) raised against a shorter peptide consisting of 11 amino acid (aa) residues of European robin Cry1b protein. The additional antibody showed identically the same immunoreactivity pattern as our original Cry1b antibody. The fact that two independent antibodies mark the same structures and cell types makes it extremely likely that the labelling detected on retinal slices is really due to Cry1b immunoreactivity and not due to some unspecific staining. As for our original gwCry1b, this second polyclonal antibody was also raised against the unique Cry1b C-terminal end in order to avoid cross-reactivity with Cry1a protein.

**Fig 3:**
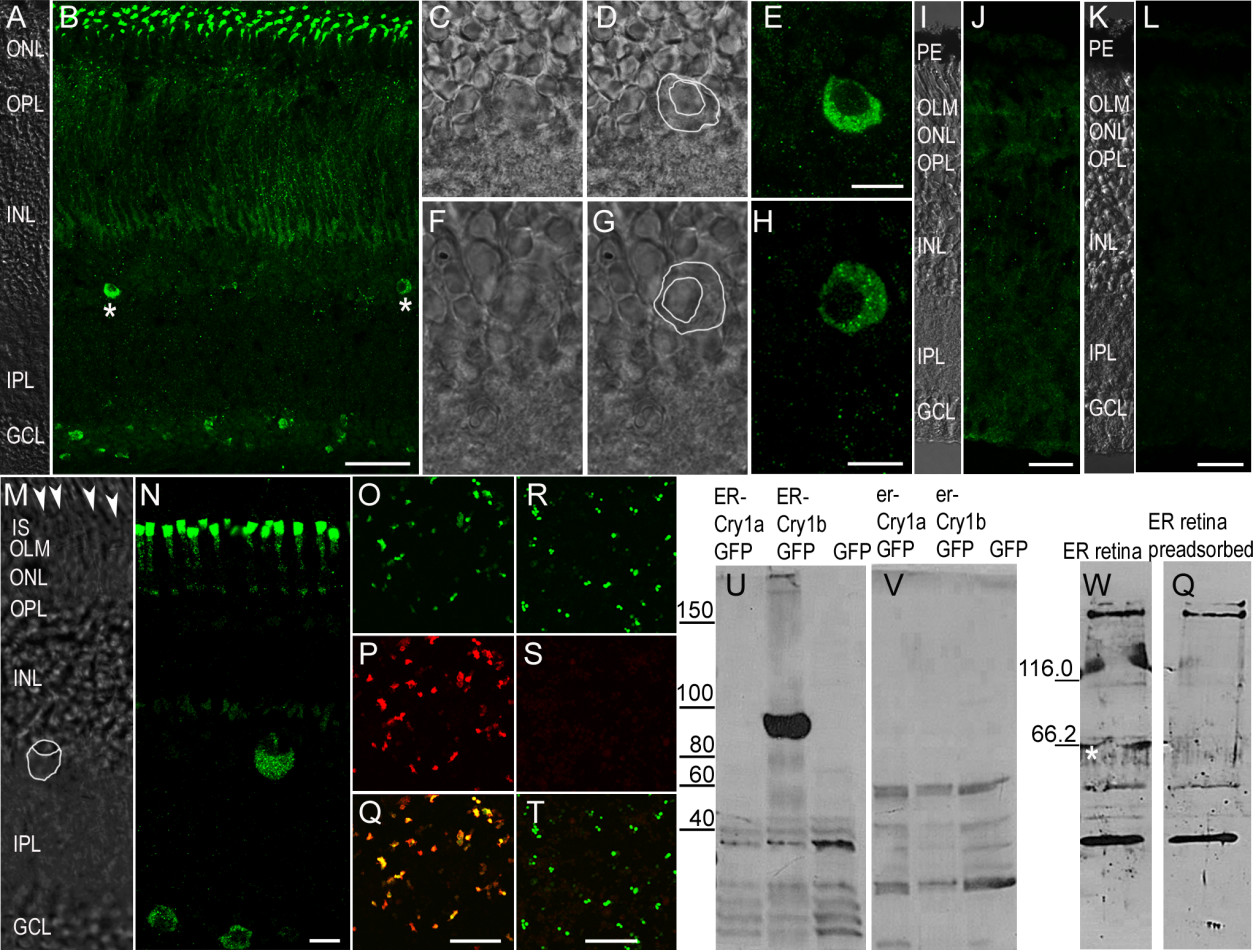
Antibodies against erCry1b confirm Cry1b expression of ganglion cells and inner segments in European robin and Northern wheatear. Immunolabelling of the erCry1b antibody in the retina of a European robin (A) revealed Cry1b expression (B) in several ganglion cells, in the photoreceptor inner segments and in few cells in the INL (B asterisks). Immunoreactive cells in the very proximal INL (C-H) were significantly bigger than surrounding bipolar cells (C, D, F, G) and showed a large cytosolic space. Immunoreactivity pattern shown in B was absent in controls with the erCry1b antibody blocked by erCry1b peptides (J) and in controls using pre-immune serum (L). In the retina of the Northern wheatear (M, N), the erCry1b antibody showed the same pattern as seen in European robins (N). Staining beneath the photoreceptor oil droplets (M arrowheads) and distal to the OLM indicated Cry1b expression in photoreceptor inner segments (M). Immunocytochemistry of N2a cells expressing erCry1b-GFP fusion protein (O green) showed that the erCry1b antibody (P red) detects erCry1b protein (Q yellow in the overlay). Immunolabelling of erCry1a-GFP expressing cells (R green) indicated that the same antibody did not detect Cry1a protein (S, T). In Western blots, the erCry1b antibody detected a band of the expected size of ~92 kDa in protein homogenates of erCry1b-GFP expressing N2a cells (U middle lane). No band at the appropriate molecular mass was seen in homogenates of erCry1a-GFP (U left lane) or GFP expressing cells (U right lane). The signal at the appropriate molecular mass was absent in negative controls after probing the blot with pre-immune serum (V). Western blots on protein homogenates from retinae of European robins incubated with the erCry1b antibody showed a band of the appropriate size ~64 kDa (W asterisk). This band was absent in controls when the blot was incubated with the pre-adsorbed antibody (Q). Molecular mass marker proteins are indicated on the left (kDa). All confocal images are maximum projections (B, J, L 12.8 μm; N, 3 μm). Images A-L were taken with identical settings. PE, pigment epithelium; IS, photoreceptor inner segments; OLM, outer limiting membrane; ONL outer nuclear layer; OPL, outer plexiform layer; INL, inner nuclear layer; IPL, inner plexiform layer; GCL, ganglion cell layer. Scale bars: A, B 50 μm; D-H, M, N 10 μm; I-L, O-T 25 μm.

Immunoreactivity of the erCry1b antibody in the retinae of European robins (Fig 3B) and Northern wheatears (Fig 3N) showed a similar pattern in ganglion cells, displaced ganglion cells and inner segments of the photoreceptors as seen for the gwCry1b antibody in pigeons. We found fundamentally the same expression pattern in (i) three Northern wheatears and two European robins investigated during the migratory season, (ii) five European robins investigated during the non-migratory season, and (iii) two European robins taken during the first week of March. Thus, we did not observe any systematic differences in Cry1b expression between the migratory and the non-migratory season. This confirmed earlier results, which we collected in the studies leading up to the generic Cry1 antibody study [46]. Here, we specifically probed for differences in Cry1 expression between birds collected in the migratory (N>10 individuals) and non-migratory (N>10 individuals) seasons [46 and unpublished data] but could not detect any systematic differences in the expression patterns.

In the ganglion cell layer of European robin and Northern wheatear retinae, the erCry1b antibody labelled fewer cells than the gwCry1b antibody labelled in the retinae of pigeons and the generic Cry1 antibody labelled in garden warblers [46]. Immuno-positive cells in the proximal INL showed typical characteristics of displaced ganglion cells [57,58], since they were considerably bigger than surrounding bipolar cells and showed a large cytosolic space (Fig 3C–3H and 3M; [46]). Staining between the photoreceptor oil droplets and the outer limiting membrane confirmed immunoreactivity in the photoreceptor inner segments. The erCry1b staining in the inner segments of photoreceptors in robins and wheatears was stronger compared to the staining found in pigeons (Fig 3N). The erCry1b immunoreactivity pattern in the ganglion cells and photoreceptor inner segments was absent in controls where the erCry1b antibody was pre-adsorbed (blocked) by erCry1b peptides (Fig 3J) and in controls where the erCry1b antibody was replaced by pre-immune serum (Fig 3L).

Immunocytochemistry on N2a cells expressing either erCry1a-GFP or erCry1b-GFP fusion protein showed that the erCry1b antibody detects erCry1b protein (Fig 3O–3Q) but not erCry1a protein (Fig 3R–3T). This was also confirmed on Western blots, where the erCry1b antibody labelled a band of the expected size of the Cry1b-GFP fusion protein (~92 kDa) in protein homogenates of erCry1b-GFP expressing N2a cells but not in homogenates of erCry1a-GFP or solely GFP expressing cells (Fig 3U). The signal at the appropriate molecular mass was absent in negative controls after probing the blot with pre-immune serum (Fig 3V) and gwCry1b antibody pre-adsorbed with the immunisation peptides (data not shown). In additional immunocytochemistry experiments (see S2 Fig), we confirmed that erCry1b in erCry1b-GFP transfected cells was not detected when the cells were incubated with the pre-immune serum and the secondary antibody or with the secondary antibody combined with the erCry1b antibody after it had been pre-adsorbed with the peptides used for immunisation. Western blots used to investigate retina lysates from European robins showed a band of the appropriate size (~64 kDa) when stained with the erCry1b antibody Fig 3W) but not when the erCry1b antibody had been incubated with the pre-adsorbed antibody (Fig 3Q). All other bands detected by the erCry1b antibody in the retina lysates in the Western blot were considered as nonspecific, as these additional bands were also present in blots incubated with the antibody pre-adsorbed with the immunisation peptide (Fig 3Q).

While working on the present paper, we cloned and sequenced the complete coding sequences of erCry1a and erCry1b from different European robins. In total, fourteen RT-PCR amplicons (eight for erCry1a and six for erCry1b) from eleven different individual birds were produced. Our sequence analyses resulted in ten variable positions (nucleotide positions 76, 77, 210, 459, 528, 546, 548, 549, 689 and 900) compared to the reference sequence in the NCBI databank (GenBank accession numbers AY585716 and AY585717, respectively; [47]). All variable nucleotides are located within the N-terminal photolyase homology region, which is identical for both erCry1a and erCry1b (see S3 Fig). At three positions (nucleotide positions 76/77, 548 and 689), these nucleotide differences are non-synonymous, resulting in a different amino acid (glutamine instead of arginine at amino acid (aa) position 26, serine instead of phenylalanine at aa183, and tryptophan instead of serine at aa230, respectively). We further identified two single nucleotide polymorphisms (SNPs) in the natural population of European Robins living in or migrating through Oldenburg, Germany. These SNPs are located at nucleotide position 177 and 651, both located within the photolyase homology region that is identical between erCry1a and erCry1b. For nucleotide 177, four of the fourteen RT-PCR amplicons bear a C instead of a T nucleotide. For nucleotide 651, we identified three RT-PCR amplicons that bear a C instead of a T nucleotide at this position. These polymorphisms are synonymous and do not affect the amino acid sequence. We are confident that our revised sequences are consistent with the native Cry1a and Cry1b nucleotide sequences in European robins since the same ten mismatches were detected in all fourteen RT-PCR amplicons from eleven different individual birds. In nine of the ten nucleotide positions, where the sequence data from our eleven birds disagreed with the NCBI databank sequences [47], our revised sequences are also in agreement with the Cry1a and Cry1b sequences reported in the only other night-migratory songbird, the garden warbler, for which a NCBI sequence is available: gwCry1a and gwCry1b (GenBank accession numbers AJ632120 and DQ838738, respectively; [46]). We can exclude cross contamination with gwCry1a and gwCry1b sequences since the gwCry1a and gwCry1b sequences consistently differed from our erCry1a and erCry1b amplicons at other locations. The corrected erCry1a and erCry1b sequences were deposited in the NCBI databank and received the GenBank accession numbers KT380948 and KT380949, respectively.

## Discussion

The reliability of immunocytochemistry results is strongly dependent on the specificity of the diagnostic antibody binding to the immunisation peptide of the focal species. A number of publications have discussed the problem of inaccurate data as a major problem in published immunocytochemical results, and consequently proposed guidelines for proper controls in immunocytochemistry [59–61]. Determining antibody specificity is especially difficult in birds since there are no knock-out animals available, which is the best specificity control in model-animals such as mice [60–62]. Without tissue from knock-out animals, it is almost impossible to conclusively exclude antibody binding at similar epitopes on proteins other than the immunisation peptide. However, we did replace the primary antibody with pre-immune serum, pre-adsorbed the antibody with its immunisation peptide, tested the antibody on cells expressing Cry1b protein, and performed Western blots followed by mass spectrometric confirmation of Cry1b presence in the detected band. Thereby, we performed sufficient (and more than required by the immunocytochemistry guidelines) controls in order to be able to as convincingly as possible demonstrate that our antibodies are very likely to bind exclusively to Cry1b protein in the retina. Furthermore, we showed that our antibodies do not detect the Cry1b homologue alternative splice product, Cry1a, and we confirmed the Cry1b immunoreactivity pattern by confirming that two different Cry1b antibodies bind to the same retinal structures. Furthermore, Niessner et al. [63] published in parallel with this paper also found Cry1b to be located in ganglion cells and displaced ganglion cells.

In the retinae of pigeons as well as two night-migratory songbird species, the European robin and the Northern wheatear, Cry1b immunoreactivity was localised in the cytoplasm of ganglion cells and displaced ganglion cells and in the photoreceptor inner segments. In Northern wheatears and European robins, the same pattern was present independent of migratory season. Expression of Cry1 protein has already been demonstrated in ganglion cells, displaced ganglion and photoreceptors cells of migratory garden warblers, but the antibody commercially available at that time did not distinguish between Cry1a and Cry1b protein [46]. The reported Cry1 immunoreactivity pattern is in line with our study: Cry1a was detected exclusively in the outer segments of UV/V cones [48] and therefore the Cry1 signal in ganglion cells, displaced ganglion cells and inner segments reported in [46] is likely to have originated from Cry1b protein.

The immuno-labelled cells detected with our diagnostic antibody were evenly distributed across the entire retina, which is in accordance with the radical-pair model [13]. In order to serve as a radical-pair-based magnetoreceptor, the cryptochrome protein should ideally be oriented consistently in a specific fashion across the retina [13,27] and must be restricted in its motion (e.g. membrane-associated [30,32,64]). Based on these anatomical requirements, photoreceptors have been discussed as the most suitable retinal cell type for harbouring magnetic-sensory molecules (e.g. [5,32,48,50]). Photoreceptor outer segments contain stacks of membranes all oriented parallel to the retinal surface, thereby providing a suitable location for cryptochrome-based magnetoreception [32]. Thus, Cry1a immunoreactivity in the photoreceptor outer segments of UV/V cones may suggest a putative magnetoreceptor role for Cry1a [48]. Another suited location in the bird’s retina that would allow for a highly ordered organisation of magnetically sensitive proteins are the cylindrical photoreceptor inner segments [32], where we detected Cry1b immunoreactivity.

We also found Cry1b protein in ganglion cells, another cell type that has been discussed as potential location for magnetoreception [46,50]. Ganglion cells do not provide an oriented cylindrical membrane structure like photoreceptor outer and inner segments, but could still harbour radical-pair-based magnetoreceptors if the magneto-sensory protein were anchored to specific portions of the cytoskeleton or cytosolically embedded membranes. Recent studies suggest that restriction of one rotational degree of freedom of a radical-pair-forming molecule is sufficient, so that every membrane-bound molecule is adequately constrained to serve as a radical-pair-based magnetoreceptor [30,32,64], and ordering might not be so important if polarised light enters the eye—as it would in nature—since polarised light would result in a photo-selective effect [5,31].

Based on the data presented here, the involvement of Cry1b in regulation of circadian activity, as shown for various cryptochromes in other vertebrates [44,45,51–55,65], is unlikely because we found Cry1b immunoreactivity to be exclusively located in the cytosol of ganglion cells, displaced ganglion cells and photoreceptors, whereas cryptochromes involved in regulating circadian activity are predominantly nuclear proteins [53,56]. We cannot, however, completely exclude that Cry1b could enter the nucleus as part of a clock-related complex in which the epitope recognised by the antibody might be hidden. In contrast, Cry2 is highly likely to be a clock protein in migratory birds due to its nuclear localisation in migratory birds [46,47,50].

Previously, the only cytosolic cryptochrome protein that had been detected in the retina of migratory birds was unspecified Cry1 [46] and Cry1a [48]. Here we show that Cry1b is also expressed in the retina of migratory birds, but its localisation distinctly differs from that of its splicing variant Cry1a. It is likely that the different retinal expression patterns of the alternative splicing products Cry1a and Cry1b are due to different retinal functions, which are likely to be controlled by their very different C-terminal domains [36,47]. It is generally assumed that the specific C-terminal regions of the multigene family are responsible for cryptochrome-specific functions [66], and that the C-terminus is essential for protein-protein interactions [67]. The photoreceptor outer segments of UV/V cones, where Cry1a has been localised, are the most likely site for cryptochrome-associated magnetoreception [32]. However, in contrast to Cry1a, which is homologous to the circadian clock protein Cry1 in mammals, Cry1b has so far only been detected in birds, suggesting bird-specific properties. To date, motion-restricted membrane binding and protein-protein interactions have not been demonstrated for any cryptochrome. To get further insight into the putative involvement of Cry1b (or any other member of the cryptochrome multigene family) in magnetoreception, crystallisation and subsequent modelling, spectroscopic measurements, and studies to unravel the signalling pathways are necessary.

From the immunocytochemical experiments reported here, we can conclude that Cry1b is expressed in the retina of migratory birds and pigeons. Different sub-cellular localisation patterns for Cry1a [48] and Cry1b detected via immunolabelling suggest different functions for these alternate splice products in birds. In migratory Northern wheatears and European robins, a similar pattern was observed throughout the year, i.e. we observed no differences in expression between the migratory season and the non-migratory season, Cry1b-positive cells were distributed across the entire retina. Cry1b is unlikely to play a role in regulation of the internal circadian clock due to its cytosolic localisation. Even though our data show that Cry1b is localised in retinal structures where it could potentially serve as a radical-pair-based magnetoreceptor, there is at present no compelling evidence indicating which of the four cryptochromes found in migratory birds—if any—is involved in light-dependent magnetoreception.

## Materials and Methods

### Animal capture, care and use

We did not kill any bird exclusively for the present study. We only used birds that had to be killed anyway for other reasons. The possibility to “use” tissue from animals which were euthanized for other experiments allowed us to keep the total number of experimental animals at a minimum. A total of 16 adult birds (i.e. aged at least 6 months) were used in this study: three Northern wheatears, ten European robins, and three pigeons. European robins (*Erithacus rubecula*) were wild caught in the vicinity of the university campus using mist nets. Capture permits were issued by the NLWKN (Niedersächsischer Landesbetrieb für Wasserwirtschaft, Küsten- und Naturschutz), reference number GB IV, D4. Northern Wheatears were bred in outdoor aviaries at the Instititute of Avian Research in Wilhelmshaven, Germany, from parents from a Norwegian population. After independency, birds were moved indoors. Animals were carefully moved in appropriate transport boxes (with water and food provided) between animal facility and laboratories. Pigeons were obtained from local breeders. Birds were handled by qualified personnel only. None of the used species are classified as an endangered species. European robins and Northern Wheatears were housed singly indoors under local photoperiodic conditions in custom built iron-free cages measuring 100x50x40cm. Pigeons were housed in an outdoor aviary measuring 300x400x200cm. The cages fulfill the EU directive specifications for holding cages. In their housing caches, the animals could move about freely. Food and water were provided *ad libitum* and complemented by mealworms. A water bath was provided once a week. In our bird facility, animal caretakers look after the birds on a daily basis. The caretakers have worked with birds for years now, and they know that if a bird is looking “puffed”, if it is unusually calm, or if the feathers are not kept in perfect condition, immediate attention is needed. Any routine treatments are done by the animal caretakers. Additionally an on-site veterinarian is present in our animal facilities. Cages and aviaries were enriched with wooden flakes/sand on the ground and three perches at different levels.

Euthanasia was normally achieved by decapitation, in order to avoid effects of injected substances on tissues of interest. If the tissue analysis for other projects required perfusion, single birds were deeply anaesthetized using an intramuscular injection of a 1:1 mix of Ketamine/Domitor (50-100mg/kg bodyweight each). Tissue was flushed transcardially using 0.9% NaCl and fixed using 4% paraformaldehyde (PFA). Euthanasia was performed by FELASA certified personnel only with >10 years of experience (Dominik Heyers). Both decapitation and transcardial perfusion conform to institutional guidelines for animal welfare and the laws on the involvement of animals in experimental research issued by the German government and represent the most commonly used techniques to obtain tissue samples for various histological analyses since decades. Approval was obtained by the Lower Saxony State animal care committee “LAVES” (Niedersächsisches Landesamt für Verbraucherschutz und Lebensmittelsicherheit), Az: 33.9-42502-04-13/1263; Az: 33.9-42502-04-11/0423; Az: 33.12-42502-04-10/0121; Az: 33.9-42502-04-12/0766.

### Cloning

Retinae from freshly prepared eyecups were vortexed for 2 min in TRIzol Reagent (Life Technologies, Carlsbad, California, USA), placed into liquid nitrogen and stored at -80°C. RNA samples were extracted according to the RNA preparation protocol for the TRIzol Reagent (Life Technologies), contaminating genomic DNA was digested with DNase I Amplification Grade (Invitrogen, Carlsbad, CA). cDNA synthesis was performed with SuperScriptIII Reverse Transcriptase (Invitrogen) according to the manufacturer’s protocol. Specific primers with restriction sites were designed to amplify the coding region of European robin (er) Cry1a (GenBank accession number AY585716) and erCry1b (GenBank accession number AY585717). erCry1a sense (5'-aaagctagcatgggggtgaacgc-3') and antisense (5'-aaaggatccgtgtaatttgtgctctgtc-3') primers and erCry1b sense (5'-aaagctagcatgggggtgaacgc-3') and antisense (5'-acgtcgacccaaaatctatccatagtatt-3') primers were used to amplify the entire respective coding regions with the RT-PCR kit GoTaq® Long PCR Master Mix (Promega, Madison, WI, USA) from total RNA of European robin retina. To amplify the coding region of garden warbler (gw) Cry1a (GenBank accession number AJ632120), we used the vector pDIA92B-His-CRY1a as a template; similarly, the amplification of the gwCry1b transcript (GenBank accession number DQ838738) was carried out using the vector pDIA92B-HisCRY1b as template [36]. The following primer pairs were used for amplification: (i) gwCry1a sense 5'aaagctagccaccatgcaccatcaccatcaccatgc-3'; gwCry1a antisense 5'-tttaagcttatttgtgctctgccgctggac-3', and (ii) gwCry1b sense 5'-aataagctttatgcaccatcaccatcacc-3' and gwCry1b antisense 5'-aatggatccgcggccgctgatccttctgatg-3’. Digested PCR products (gwCry1a: *NheI/HindIII*; gwCry1b: *HindIII/BamHI;* erCry1a: *NheI/BamHI*; erCry1b: *NheI/SalI*) were first subcloned into pGEM T easy vector (Promega, Madison, WI, USA) and, where necessary, subsequently cloned into the expression vector pTurboGFP-N (Evrogen, Moscow, Russia) according to the protocol for the Rapid DNA Dephos & Ligation Kit (Roche Diagnostics, Mannheim, Germany). Cloned fragments were subjected to sequencing and sequence analyses, alignments of the reference sequences were carried out using the ClustalW server from the European Bioinformatics Institute.

### Expression of Cry1a and Cry1b protein

For immunocytochemical labelling of gwCry1a-GFP, erCry1a-GFP, gwCry1b-GFP, erCry1b-GFP and GFP expressing cells, mammalian neuroblastoma (N2a) cells were plated on poly-L-Lysine-coated coverslips to 90% confluence in 24-well culture dishes for transient transfection. Cells were transfected in Opti-MEM medium (Invitrogen) with 1 μg of plasmid DNA following the manufacturers protocol for Lipofectamine2000 transfection reagent (Invitrogen) using 2 μl Lipofectamine 2000 reagent per μg DNA. Six hours after transfection, the DNA/lipofectamine suspension was replaced by DMEM medium (Invitrogen) including 10% fetal calf serum (FCS) and Gibco Antibiotic-Antimycotic according to the manufacturer’s protocol (Life Technologies). After 24 hours, coverslips were fixed for 15 minutes in 4% PFA.

For Western blot analysis using the erCry1b antibody, N2a cells were transfected in poly-L-Lysine-coated 10 cm petri dishes using appropriate volumes of reagents as described above. For Western blot analysis using the gwCry1b antibody, gwCry1a and gwCry1b protein without a GFP tag were recombinantly expressed in a Sf9/baculovirus expression system as previously described [36].

### Primary antibodies

A polyclonal antibody detecting garden warbler Cry1b (gwCry1b) protein was raised in rabbits (Pineda Antibody Service, Berlin, Germany). Antibodies were generated against a synthetic peptide representing 17 amino acids (aa 571–587; C-RGSPNPCNYGKPDKTSE-N) corresponding to the C-terminal end of the garden warbler Cry1b protein sequence (GenBank accession number ABH03083). To produce an antibody detecting European robin Cry1b (erCry1b) protein, rabbits were immunised with a peptide consisting of 11 aa residues (577–587; C-CNYGKPDKTSK-N) corresponding to the C-terminal end of the European robin Cry1b protein sequence (GenBank accession number AAW48291). A goat polyclonal OPN1SW antibody (N-20): sc-14363 (Santa Cruz Biotechnology Inc., Santa Cruz, CA, USA) mapping to the amino (N)-terminus of OPN1SW of human origin was used to detect outer segments of UV/V cones [48].

### Immunocytochemistry of transfected cells

For immunocytochemistry, transfected cells were rinsed in PBS, incubated in 0.1% Triton X100/PBS for 30 minutes, and blocked with 10% donkey serum (Sigma, Deisenhofen, Germany) in PBS for one hour. A primary antibody was used in a 1:500 dilution for gwCry1b and in a 1:2000 dilution for erCry1b at 4°C overnight. Incubation with the secondary antibody Alexa555-donkey-anti-rabbit (Invitrogen, Karlsruhe, Germany) was performed in 1:500 dilutions in PBS for two hours at room temperature. Cover slips were rinsed in PBS and water before being mounted with Vectashield Mounting medium (Vector Laboratories Inc., Burlingame, CA, USA).

### Western blot analysis

After transfection, the erCry1a-GFP, erCry1b-GFP and GFP expressing N2a cells were harvested, centrifuged and the cell pellet was dissolved in lysis buffer containing 150 mM NaCl, 1% Nonident P 40, 50 mM Tris HCL pH 8,0 and 1 cOmplete ULTRA® Tablet per 50ml according to the manufacturer’s protocol (Roche Diagnostics, Mannheim, Germany). European robin retinae were treated with Roti®-Load 1 loading dye (one third of sample volume, Carl Roth, Karlsruhe, Germany) and boiled for 5 min. Homogenates of pigeon retinae and of gwCry1a and gwCry1b protein (recombinantly expressed in an Sf9/baculovirus expression system as previously described [36]) were resuspended in gel-loading buffer (Laemmli, 1970).

Proteins (50 μg per lane) were separated by SDS-PAGE on 8–10% gradient gels (gwCry1b antibody) or 8% gels (erCry1b antibody) and transferred to a nitrocellulose membrane (Optitran BA-S 85, Schleicher Schuell, Dassel, Germany) or transferred to a polyvinylidene fluoride membrane when probing with the erCry1b antibody. Unspecific binding sites were blocked for one hour (gwCry1b antibody: 5% powdered milk in TBS-Tween at 37°C; erCry1b antibody: Roti®Block (Carl Roth, Karlsruhe, Germany) 1:10 with aqua bidest at RT).

Incubation with the primary antibodies (gwCry1b antibody: 1:500 in TBS-Tween: 20 mM Tris/HCl, 150 mM NaCl, 0.2% Tween-20; erCry1b antibody: 1:500 in Roti®Block 1:10 with TBS-Tween) was carried out overnight at 4°C. After washing in TBS-Tween, immunoreactive protein was visualised with horseradish peroxidase-conjugated goat anti-rabbit IgG (gwCry1b: 1:3000 in TBS-Tween with 2% powdered milk; BioRad Laboratories, Munich, Germany; erCry1b antibody: 1:5000 in Roti®Block, 1:10 with TBS-Tween) using the Pierce enhanced chemiluminescence detection system (Pierce ECL; Thermo Fisher Scientific, Rockford, IL, USA), following the instructions given by the manufacturer.

To test the specificity of the erCry1b antibody, PVDF membranes (50 μg of protein per lane) were incubated either with the pre-immune serum (1:5000 in Roti®Block, 1:10 with TBS-Tween, pH 7.4) or with erCry1b antibody pre-adsorbed 1:50 with its accordant peptide (1:500 in Roti®Block 1:10 with TBS-Tween). To examine unspecific tissue reactions of the gwCry1b antibody, the bound gwCry1b antibody was removed under shaking in two washing steps (1^st^ washing in 1% SDS, 10 mM Tris/HCl, pH 8.8, 10 mM β-mercaptoethanol; 2^nd^ washing in 1% SDS, 100 mM sodium citrate, pH 3.0, 10 mM β-mercaptoethanol) for 1 hour at 37°C. After washing in TBS-Tween, blots were blocked and re-probed with the pre-immune serum or the gwCry1b antibody (1:500) pre-adsorbed for 4 hours with the immunisation peptide (1 mg/ml PBS stock solution 1:50).

### Protein identification by nanoLC-ESI-MS/MS

SDS-PAGE separated protein bands for subsequent mass spectrometric analysis were selected by comparison to positions detected by the corresponding Western blot. Respective bands were excised, cut into smaller pieces and subjected to in-gel digest as described before [68]. Separation of generated tryptic peptides was performed with an Ultimate3000 nanoRSLC system (Thermo Scientific, Germering, Germany) equipped with a trap column (PepMap nanoTrap, C18 100Å, 3 μm bead size, 75 μm inner diameter, 2 cm length; Thermo Scientific) and a 15 cm analytical column (C18 100Å, 2 μm bead size, 75 μm inner diameter, 15 cm length; Thermo Scientific). The following linear gradient of increasing acetonitrile concentration was applied using solvent A (0.1% FA) and solvent B (80% acetonitrile, 0.1% FA): 0–12 min 4% B, 12–120 min 4–50% B, 120–130 min 50–95% B, 130–135 min 95% B. The eluent was continuously analysed by an online coupled electrospray-ionisation ion-trap mass spectrometer (amazon ETD; Bruker Daltonik GmbH, Bremen, Germany) operating as follows: For electrospray ionization, a distal coated fused SilicaTip (NewObjective, Woburn, USA) with an inner diameter of 10 μm was used at a voltage of 1,600 V. The MS method consisted of a full MS scan (mass range 300–1,500 m/z) with subsequent MS/MS of the ten most intense MS peaks (successive exclusion after 1 spectrum for 0.2 min). Protein identification was performed with ProteinScape (version 3.1, Bruker Daltonik) on a Mascot server (version 2.3, Matrix Science, London, UK) against the protein sequences of the *Columba livia* genome downloaded from the NCBI database (status june 2013). The database was supplemented with the known cryptochrome sequences of *Sylvia borin* (gi45535501, gi110962429, gi52699557, gi260586482), *Erithacus rubecula* (gi57233429, gi57233431, gi54780882), *Taeniopygia guttata* (gi449492091) and *Gallus gallus* (gi45383636, gi45383642, gi31321921). A target-decoy strategy with a false discovery rate of <1.0% was applied, using the following setting: enzyme, trypsin; fixed modification, carbamidomethyl (C); variable modification, oxidation (M); peptide mass tolerance for MS and MS/MS, 0.4 Da (monoisotopic); significance threshold, p<0.05.

### Immunohistochemistry

For immunocytochemistry, birds were anaesthetised and perfused transcardially with saline followed by 4% paraformaldehyde (PFA) in phosphate buffered saline (PBS). Eyes were prepared by cornea dissection followed by removal of the lens and vitreous bodies. After preparation, eyecups were post-fixed in 4% PFA/PBS for 20 minutes and washed in PBS. Tissue adaptation to cryoprotectant solution was performed overnight at 4°C in 30% saccharose solution in 0.1 M PBS and embedded in cryoblock at -20°C. Vertical retinal sections of 20 μm were cut on a cryostat, collected on gelatinised superfrost coverslips (Carl Roth, Karsruhe, Germany) and heat-fixed at 37°C for three hours. When analysing immunoreactivity of the outer segments with the gwCry1b antibody, retinal pigment epithelium was bleached by immersing the retinal slices for 30 minutes in 5 ml 1.8% NaCl, 4 ml 30% H2O2, 1 ml H2O and one drop of ammonia solution to make the outer segments visible for microscopy. After washing (gwCry1b antibody: PBS; erCry1b antibody: PBS containing 0,3% Triton-X-100), cryosections were blocked with 10% normal donkey serum (NDS, Sigma, Deisenhofen, Germany) for one hour (gwCry1b antibody: in PBS containing 0.3% Tween and 2% BSA; erCry1b antibody: in PBS containing 0,3% Triton-X-100) and then incubated with the primary antibody (gwCry1b 1:500, erCry1b 1:2000; sc-14363 1:500) in PBS-Tween (erCry1b antibody: PBS containing 0,3% Triton-X-100) containing 2% NDS at 4°C overnight. After several washes, slices were incubated with an appropriate secondary antibody coupled to the fluorescent dyes Alexa555 and Alexa488 (Invitrogen, Karlsruhe, Germany) for two hours. The secondary antibody was diluted 1:500 in PBS-Tween (erCry1b antibody: in PBS containing 0,3% Triton-X-100) containing 2% NDS. In control experiments, the Cry1b antibody was pre-adsorbed with the appropriate inhibitory peptide (ratio 1:10) for four hours before applying the primary antibody on the slices. To further examine unspecific staining caused by other antibodies in the serum, the Cry1b antibody was replaced with pre-immune serum taken before immunizing the animals. After final washes the slices were mounted with Vectashield or Roti® Mount-FluorCare (Carl Roth GmbH Karlsruhe, Germany).

### Confocal microscopy

Confocal fluorescent micrographs of retinae and N2a cells were taken using the 488 and 555 nm lines with a Leica TCS SP5 gated STED microscope. Scanning was performed with an oil-immersion HCX PL APO 100.0x1.40 OIL objective at a resolution of either 1024x1024, 2048x2048 or 4096x4096 pixels. Images are presented as projections sof z-stacks of 1–27 μm. Images were processed in brightness and contrast and superimposed using either ImageJ (NIH, Bethesda, MD) or Photoshop CS3 Extended (Adobe, San Jose, CA).

## Supporting Information

S1 Fig gwCry1b-GFP expressing N2a cells do not show cross-reactivity of pre-immune serum and secondary antibody. **A:** Immunocytochemistry of N2a cells expressing gwCry1b-GFP fusion protein (green) showed that the gwCry1b antibody (red) detect gwCry1b protein (yellow in the overlay). **B:** Immunolabelling of gwCry1a-GFP expressing cells (green) indicated that the same antibody (red) did not detect gwCry1a protein. **C:** Labelling was absent in controls with pre-immune serum and **D:** in controls with omitted primary antibody.
(TIF)

S2 Fig No cross-reactivity of pre-immune serum and pre-adsorbed erCry1b antibody on erCry1b-GFP expressing N2a cells. **A:** Immunocytochemistry of N2a cells expressing erCry1b-GFP fusion protein (green) showed that the erCry1b antibody (red) detects erCry1b protein (yellow in the overlay). **B:** Immunolabelling of erCry1a-GFP expressing cells (green) indicated that the same antibody (red) did not detect Cry1a protein. **C:** Labelling was absent in controls with pre-immune serum and **D:** in controls with gwCry1b antibody blocked by the accordant gwCry1b peptides.
(TIF)

S3 Fig Alignment of Cry1a and Cry1b coding sequence amplified from the retinae of European robins and the corresponding NCBI databank sequences. Partial sequence alignment of RT-PCR amplified sequences from retinae of European robin (shown in capitalised letters) revealed ten mismatches (red), when compared with the deposited NCBI databank sequences (shown in uncapitalised letters) of European robin Cry1a (AY585716) and Cry1b (AY585717). The additional punctually appearing mismatches (blue) are most probably polymerase and/or sequencing mistakes.
(PDF)

S1 Table Proteins identified from SDS-PAGE separated protein band corresponding to the Western blot detected Cry1b. (XLSX)

S2 Table Detected peptides of the Cry1b protein of *Sylvia borin*. ^a^ Detected modifications: Cm, carbamidomethyl (C); Ox, oxidation (M).
(XLSX)
