# Supplementary figures and images for: Localisation of the Putative Magnetoreceptive Protein Cryptochrome 1b in the Retinae of Migratory Birds and Homing Pigeons
Source: PLoS One. 2016 Mar 8;11(3):e0147819. doi: 10.1371/journal.pone.0147819 (PMC4783096; doi:10.1371/journal.pone.0147819)

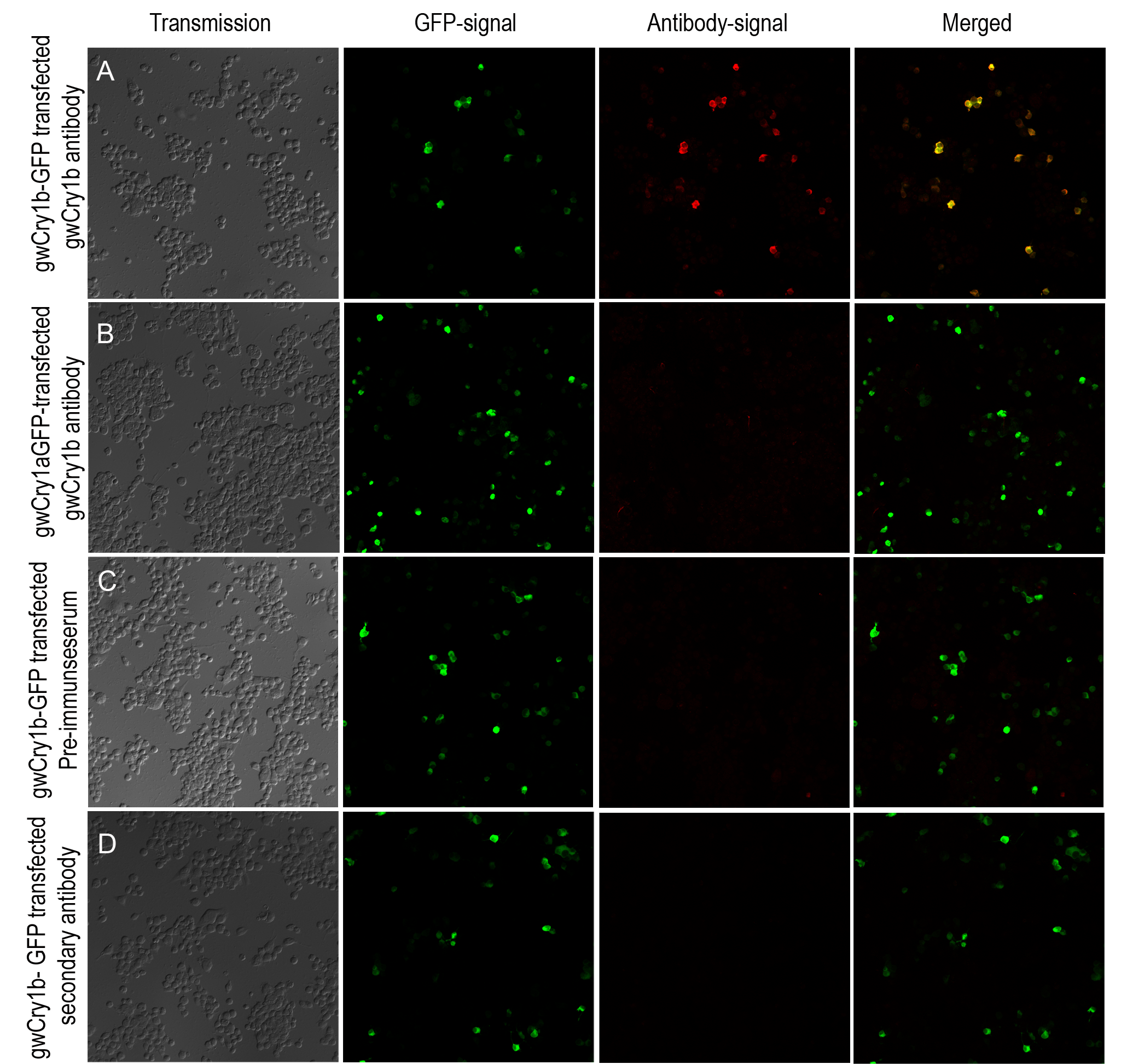

Supplement: S1 Fig — A: Immunocytochemistry of N2a cells expressing gwCry1b-GFP fusion protein (green) showed that the gwCry1b antibody (red) detect gwCry1b protein (yellow in the overlay). B: Immunolabelling of gwCry1a-GFP expressing cells (green) indicated that the same antibody (red) did not detect gwCry1a protein. C: Labelling was absent in controls with pre-immune serum and D: in controls with omitted primary antibody. (TIF) [file pone.0147819.s001.tif]

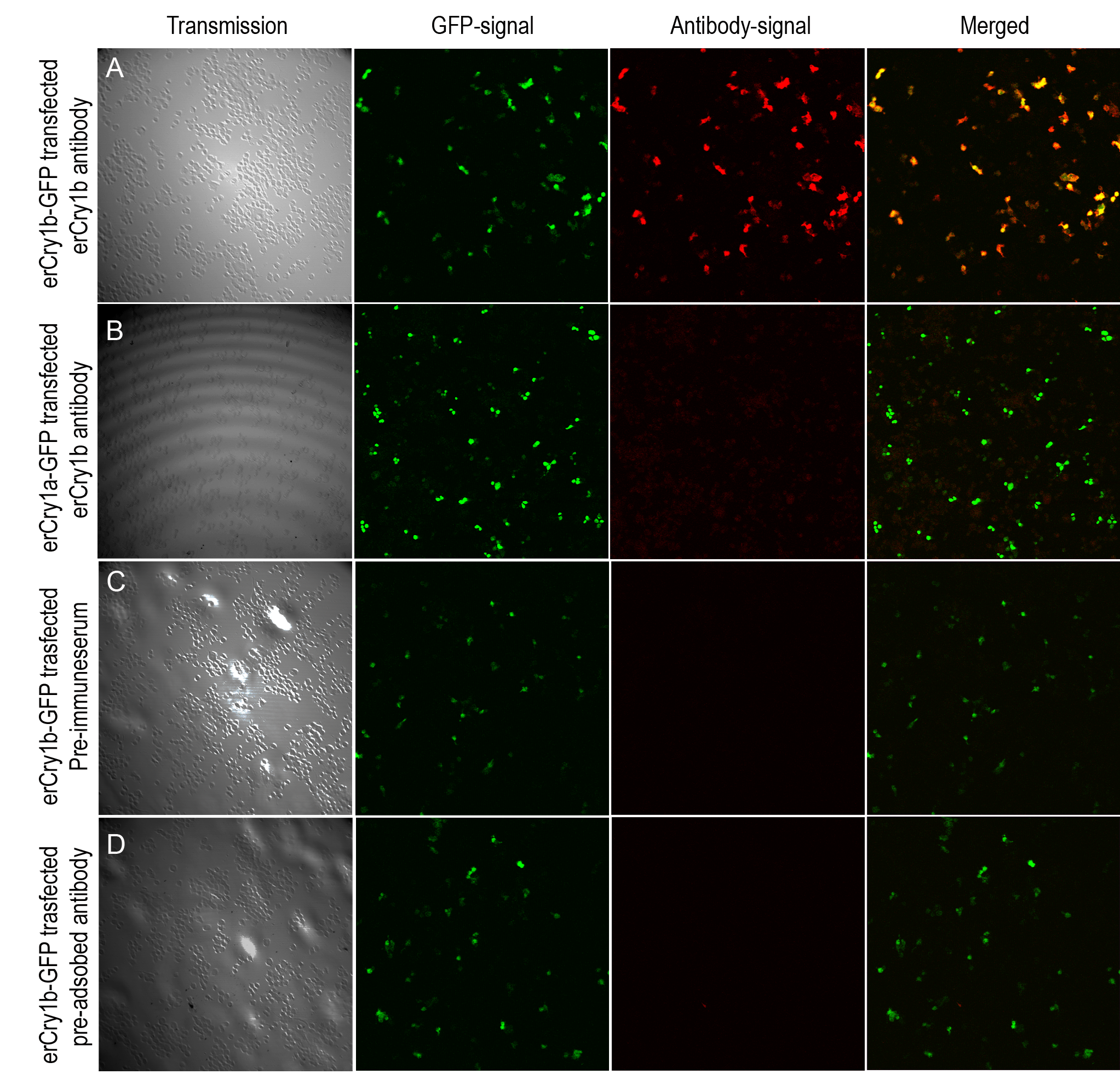

Supplement: S2 Fig — A: Immunocytochemistry of N2a cells expressing erCry1b-GFP fusion protein (green) showed that the erCry1b antibody (red) detects erCry1b protein (yellow in the overlay). B: Immunolabelling of erCry1a-GFP expressing cells (green) indicated that the same antibody (red) did not detect Cry1a protein. C: Labelling was absent in controls with pre-immune serum and D: in controls with gwCry1b antibody blocked by the accordant gwCry1b peptides. (TIF) [file pone.0147819.s002.tif]
