# Supplementary material for: Localisation of the Putative Magnetoreceptive Protein Cryptochrome 1b in the Retinae of Migratory Birds and Homing Pigeons
Source: PLoS One. 2016 Mar 8;11(3):e0147819. doi: 10.1371/journal.pone.0147819 (PMC4783096; doi:10.1371/journal.pone.0147819)
Supplement: S3 Fig — Partial sequence alignment of RT-PCR amplified sequences from retinae of European robin (shown in capitalised letters) revealed ten mismatches (red), when compared with the deposited NCBI databank sequences (shown in uncapitalised letters) of European robin Cry1a (AY585716) and Cry1b (AY585717). The additional punctually appearing mismatches (blue) are most probably polymerase and/or sequencing mistakes. (PDF) [file pone.0147819.s003.pdf]

erCry1a Accession number KT380948

```
AY585716      atgggggtgaacgccgtgcactggttccgcaaggggctgcggctccacgacaacccggcg    60
87yellow      ATGGGGGTGAACGCCGTGCACTGGTTCCGCAAGGGGCTGCGGCTCCACGACAACCCGGCG    60
55yellow      ATGGGGGTGAACGCCGTGCACTGGTTCCGCAAGGGGCTGCGGCTCCACGACAACCCGGCG    60
16white       ATGGGGGTGAACGCCGTGCACTGGTTCCGCAAGGGGCTGCGGCTCCACGACAACCCGGCG    60
3brown        ATGGGGGTGAACGCCGTGCACTGGTTCCGCAAGGGGCTGCGGCTCCACGACAACCCGGCG    60
18brown       ATGGGGGTGAACGCCGTGCACTGGTTCCGCAAGGGGCTGCGGCTCCACGACAACCCGGCG    60
27brown       ATGGGGGTGAACGCCGTGCACTGGTTCCGCAAGGGGCTGCGGCTCCACGACAACCCGGCG    60
20pink        ATGGGGGTGAACGCCGTGCACTGGTTCCGCAAGGGGCTGCGGCTCCACGACAACCCGGCG    60
30pink        ATGGGGGTGAACGCCGTGCACTGGTTCCGCAAGGGGCTGCGGCTCCACGACAACCCGGCG    60
Consensus     ATGGGGGTGAACGCCGTGCACTGGTTCCGCAAGGGGCTGCGGCTCCACGACAACCCGGCG    60
              ************************************************************

AY585716      ctgcgggaatgcatcaggggcgccgacacggtgcgctgcgtctacatcctggacccctgg    120
87yellow      CTGCGGGAATGCATCCAGGGCGCCGACACGGTGCGCTGCGTCTACATCCTGGACCCCTGG    120
55yellow      CTGCGGGAATGCATCCAGGGCGCCGACACGGTGCGCTGCGTCTACATCCTGGACCCCTGG    120
16white       CTGCGGGAATGCATCCAGGGCGCCGACACGGTGCGCTGCGTCTACATCCTGGACCCCTGG    120
3brown        CTGCGGGAATGCATCCGGGGCGCCGACACGGTGCGCTGCGTCTACATCCTGGACCCCTGG    120
18brown       CTGCGGGAATGTATCCAGGGCGCCGACACGGTGCGCTGCGTCTACATCCTGGACCCCTGG    120
27brown       CTGCGGGAATGCATCCAGGGCGCCGACACGGTGCGCTGCGTCTACATCCTGGACCCCTGG    120
20pink        CTGCGGGAATGCATCCAGGGCGCCGACACGGTGCGCTGCGTCTACATCCTGGACCCCTGG    120
30pink        CTGCGGGAATGCATCCAGGGCGCCGACACGGTGCGCTGCGTCTACATCCTGGACCCCTGG    120
Consensus     CTGCGGGAATGCATCCAGGGCGCCGACACGGTGCGCTGCGTCTACATCCTGGACCCCTGG    120
              *********** ***  *******************************************

AY585716      ttcgccggctcctccaacgtgggcatcaacaggtggcgattcctgcttcagtgtcttgaa    180
87yellow      TTCGCCGGCTCCTCCAACGTGGGCATCAACAGGTGGCGATTCCTGCTTCAGTGTCTTGAA    180
55yellow      TTCGCCGGCTCCTCCAACGTGGGCATCAACAGGTGGCGATTCCTGCTTCAGTGTCTCGAA    180
16white       TTCGCCGGCTCCTCCAACGTGGGCATCAACAGGTGGCGATTCCTGCTTCAGTGTCTCGAA    180
3brown        TTCGCCGGCTCCTCCAACGTGGGCATCAACAGGTGGCGATTCCTGCTTCAGTGTCTTGAA    180
18brown       TTCGCCGGCTCCTCCAACGTGGGCATCAACAGGTGGCGATTCCTGCTTCAGTGTCTTGAA    180
27brown       TTCGCCGGCTCCTCCAACGTGGGCATCAACAGGTGGCGATTCCTGCTTCAGTGTCTTGAA    180
20pink        TTCGCCGGCTCCTCCAACGTGGGCATCAACAGGTGGCGATTCCTGCTTCAGTGTCTTGAA    180
30pink        TTCGCCGGCTCCTCCAACGTGGGCATCAACAGGTGGCGATTCCTGCTTCAGTGTCTTGAA    180
Consensus     TTCGCCGGCTCCTCCAACGTGGGCATCAACAGGTGGCGATTCCTGCTTCAGTGTCTTGAA    180
              ******************************************************** ***

AY58571       gatcttgatgccaatctacggaaactgaactcacgcttgtttgttatccgtggacagcca    240
87yellow      GATCTTGATGCCAATCTACGGAAACTGAATTCACGCTTGTTTGTTATCCGTGGACAGCCA    240
55yellow      GATCTTGATGCCAATCTACGGAAACTGAATTCACGCTTGTTTGTTATCCGTGGACAGCCA    240
16white       GATCTTGATGCCAATCTACGGAAACTGAATTCACGCTTGTTTGTTATCCGTGGACAGCCA    240
3brown        GATCTTGATGCCAATCTACGGAAACTGAATTCACGCTTGTTTGTTATCCGTGGACAGCCG    240
18brown       GATCTTGATGCCAATCTACGGAAACTGAATTCACGCTTGTTTGTTATCCGTGGACAGCTA    240
27brown       GATCTTGATGCCAATCTACGGAAACTGAATTCACGCTTGTTTGTTATCCGTGGACAGCCA    240
20pink        GATCTTGATGCCAATCTACGGAAACTGAATTCACGCTTGTTTGTTATCCGTGGACAGCCA    240
30pink        GATCTTGATGCCAATCTACGGAAACTGAATTCACGCTTGTTTGTTATCCGTGGACAGCCA    240
Consensus     GATCTTGATGCCAATCTACGGAAACTGAATTCACGCTTGTTTGTTATCCGTGGACAGCCA    240
              ***************************** ****************************

AY585716      gcagatgttttccccaggctttttaaggaatggaacattgcaaagctttctattgaatat    300
87yellow      GCAGATGTTTTCCCCAGGCTTTTTAAGGAATGGAACATTGCAAAGCTTTCTATTGAATAT    300
55yellow      GCAGATGTTTTCCCCAGGCTTTTTAAGGAATGGAACATTGCAAAGCTTTCTATTGAATAT    300
16white       GCAGATGTTTTCCCCAGGCTTTTTAAGGAATGGAACATTGCAAAGCTTTCTATTGAATAT    300
3brown        GCAGATGTTTTCCCCAGGCTTTTTAAGGAATGGAACATTGCAAAGCTTTCTATTGAATAT    300
18brown       GCAGATGTTTTCCCCAGGCTTTTTAAGGAATGGAACATTGCAAAGCTTTCTATTGAATAT    300
27brown       GCAGATGTTTTCCCCAGGCTTTTTAAGGAATGGAACATTGCAAAGCTTTCTATTGAATAT    300
20pink        GCAGATGTTTTCCCCAGGCTTTTTAAGGAATGGAACATTGCAAAGCTTTCTATTGAATAT    300
30pink        GCAGATGTTTTCCCCAGGCTT-TTAAGGAATGGAACATTGCAAAGCTTTCTATTGAATAT    299
Consensus     GCAGATGTTTTCCCCAGGCTTTTTAAGGAATGGAACATTGCAAAGCTTTCTATTGAATAT    300
              ********************* **************************************

AY585716      gattctgaaccatttgggaaggagagagatgcagccatcaagaagctggccagtgaagct    360
87yellow      GATTCTGAACCATTTGGGAAGGAGAGAGATGCAGCCATCAAGAAGCTGGCCAGTGAAGCT    360
55yellow      GATTCTGAACCATTTGGGAAGGAGGGAGATGCAGCCATCAAGAAGCTGGCCAGTGAAGCT    360
16white       GATTCTGAACCATTTGGGAAGGAGAGAGATGCAGCCATCAAGAAGCTGGCCAGTGAAGCT    360
3brown        GATTCTGAACCATTTGGGAAGGAGAGAGATGCAGCCATCAAGAAGCTGGCCAGTGAAGCT    360
18brown       GATTCTGAACCATTTGGGAAGGAGAGAGATGCAGCCATCAAGAAGCTGGCCAGTGAAGCT    360
27brown       GATTCTGAACCATTTGGGAAGGAGAGAGATGCAGCCATCAAGAAGCTGGCCGGTGAAGCT    360
20pink        GATTCTGAACCATTTGGGAAGGAGAGAGATGCAGCCATCAAGAAGCTGGCCAGTGAAGCT    360
30pink        GATTCTGAACCATTTGGGAAGGAGAGAGATGCAGCCATCAAGAAGCTGGCCAGTGAAGCT    359
Consensus     GATTCTGAACCATTTGGGAAGGAGAGAGATGCAGCCATCAAGAAGCTGGCCAGTGAAGCT    360
              ************************ ************************** ********
```

```
AY585716     ggagtggaggtcattgttcggatttctcatacattgtatgacctagacaaaatcatagaa   420
87yellow     GGAGTGGAGGTCATTGTTCGGATTTCTCATACATTGTATGACCTAGACAAAATCATAGAA   420
55yellow     GGAGTGGAGGTCATTGTTCGGATTTCTCATACACTGTATGACCTAGACAAAATCATAGAA   420
16white      GGAGTGGAGGTCATTGTTCGGATTTCTCATACATTGTATGACCTAGACAAAATCATAGAA   420
3brown       GGAGTGGAGGTCATTGTTCGGATTTCTCATACATTGTATGACCTAGACAAAATCATAGAA   420
18brown      GGAGTGGAGGTCATTGTTCGGATTTCTCATACATTGTATGACCTAGACAAAATCATAGAA   420
27brown      GGAGTGGAGGTCATTGTTCGGATTTCTCATACATTGTATGACCTAGACAAAATCATAGAA   420
20pink       GGAGTGGAGGTCATTGTTCGGATTTCTCATACATTGTATGACCTAGACAAAATCATAGAA   420
30pink       GGAGTGGAGGTCATTGTTCGGATTTCTCATACATTGCATGACCTAGACAAAATCATAGAA   419
Consensus    GGAGTGGAGGTCATTGTTCGGATTTCTCATACATTGTATGACCTAGACAAAATCATAGAA   420
             ********************************* ** ***********************

AY585716     ttaaatgggggacagcctcctcttacttacaagcgattccagaccctaattagtagaatg   480
87yellow     TTAAATGGGGGACAGCCTCCTCTTACTTACAAGCGATTTCAGACCCTAATTAGTAGAATG   480
55yellow     TTAAATGGGGGACAGCCTCCTCTTACTTACAAGCGATTTCAGACCCTAATTAGTAGAATG   480
16white      TTAAATGGGGGACAGCCTCCTCTTACTTACAAGCGATTTCAGACCCTAATTAGTAGAATG   480
3brown       TTAAATGGGGGACAGCCTCCTCTTACTTACAAGCGATTTCAGACCCTAATTAGTAGAATG   480
18brown      TTAAATGGGGGACAGCCTCCTCTTACTTACAAGCGATTTCAGACCCTAATTAGTAGAATG   480
27brown      TTAAATGGGGGACAGCCTCCTCTTACTTACAAGCGATTTCAGACCCTAATTAGTAGAATG   480
20pink       TTAAATGGGGGACAGCCTCCTCTTACTTACAAGCGATTTCAGACCCTAATTAGTAGAATG   480
30pink       TTAAATGGGGGACAGCCTCCTCTTACTTACAAGCGATTTCAGACCCTAATTAGTAGAATG   479
Consensus    TTAAATGGGGGACAGCCTCCTCTTACTTACAAGCGATTTCAGACCCTAATTAGTAGAATG   480
             ************************************** *********************

AY585716     gaacccctggagatgcctgtggagactataaccccagaagtaatgaagaaatgtactact   540
87yellow     GAACCCCTGGAGATGCCTGTGGAGACTATAACCCCAGAAGTAATGAAAAAATGTACTACT   540
55yellow     GAACCCCTGGAGATGCCTGTGGAGACTATAACCCCAGAAGTAATGAAAAAATGTACTACT   540
16white      GAACCCCTGGAGATGCCTGTGGAGACTATAACCCCAGAAGTAATGAAAAAATGTACTACT   540
3brown       GAACCCCTGGAGATGCCTGTGGAGACTATAACCCCAGAAGTAATGAAAAAATGTACTACT   540
18brown      GAACCCCTGGAGATGCCTGTGGAGACTATAACCCCAGAAGTAATGAAAAAATGTACTACT   540
27brown      GAACCCCTGGAGATGCCTGTGGAGACTATAACCCCAGAAGTAATGAAAAAATGTACTACT   540
20pink       GAACCCCTGGAGATGCCTGTGGAGACTATAACCCCAGAAGTAATGAAAAAATGTACTACT   540
30pink       GAACCCCTGGAGATGCCTGTGGAGACTATAACCCCAGAAGTAATGAAAAAATGTACTACT   539
Consensus    GAACCCCTGGAGATGCCTGTGGAGACTATAACCCCAGAAGTAATGAAAAAATGTACTACT   540
             *********************************************** ************

AY585716     ccagtcttcgatgaccatgatgagaaatacggtgtgccatcacttgaagagctgggcttt   600
87yellow     CCAGTTTCTGATGACCATGATGAGAAATACGGTGTGCCATCACTTGAAGAGCTGGGCTTT   600
55yellow     CCAGTTTCTGATGACCATGATGAGAAATACGGTGTGCCATCACTTGGAGAGCTGGGCTTT   600
16white      CCAGTTTCTGATGACCATGATGAGAAATACGGTGTGCCATCACTTGAAGAGCTGGGCTTT   600
3brown       CCAGTTTCTGATGACCATGATGAGAAATACGGTGTGCCATCACTTGAAGAGCTGGGCTTT   600
18brown      CCAGTTTCTGATGACCATGATGAGAAATACGGTGTGCCATCACTTGAAGAGCTGGGCTTT   600
27brown      CCAGTTTCTGATGACCATGATGAGAAATACGGTGTGCCATCACTTGAAGAGCTGGGCTTT   600
20pink       CCAGTTTCTGATGACCATGATGAGAAATACGGTGTGCCATCACTTGAAGAGCTGGGCTTT   600
30pink       CCAGTTTCTGATGACCATGATGAGAAATACGGTGTGCCATCACTTGAAGAGCTGGGCTTT   599
Consensus    CCAGTTTCTGATGACCATGATGAGAAATACGGTGTGCCATCACTTGAAGAGCTGGGCTTT   600
             ***** *  ************************************* *************

AY585716     gacacagatggtctgccttctgcagtatggccagggggagaaactgaagctctcacacgc   660
87yellow     GACACAGATGGTCTGCCTTCTGCAGTATGGCCAGGGGGAGAAACTGAAGCTCTCACACGC   660
55yellow     GACACAGATGGTCTGCCTTCTGCAGTATGGCCAGGGGGAGAAACTGAAGCTCTCACACGC   660
16white      GACACAGATGGTCTGCCTTCTGCAGTATGGCCAGGGGGAGAAACTGAAGCTCTCACACGC   660
3brown       GACACAGATGGTCTGCCTTCTGCAGTATGGTCAGGGGGAGAAACTGAAGCTCTCACACGC   660
18brown      GACACAGATGGTCTGCCTTCTGCAGTATGGCCAGGGGGAGAAACTGAAGCTCTCACACGC   660
27brown      GACACAGATGGTCTGCCTTCTGCAGTATGGCCAGGGGGAGAAACTGAAGCTCTCACACGC   660
20pink       GACACAGATGGTCTGCCTTCTGCAGTATGGCCAGGGGGAGAAACTGAAGCTCTCACACGC   660
30pink       GACACAGATGGTCTGCCTTCTGCAGTATGGCCAGGGGGAGAAACTGAAGCCCTCACACGC   659
Consensus    GACACAGATGGTCTGCCTTCTGCAGTATGGCCAGGGGGAGAAACTGAAGCTCTCACACGC   660
             ****************************** ****************** **********

AY585716     ttagaaagacatttagaacgaaaggcttcggtagcaaactttgaaagaccacggatgaat   720
87yellow     TTAGAAAGACATTTAGAACGAAAGGCTTGGGTAGCAAACTTTGAAAGACCACGGATGAAT   720
55yellow     TTAGAAAGACATTTAGAACGAAAGGCTTGGGTAGCAAACTTTGAAAGACCACGGATGAAT   720
16white      TTAGAAAGACATTTAGAACGAAAGGCTTGAGTAGCAAACTTTGAAAGACCACGGATGAAT   720
3brown       TTAGAAAGACATTTAGAACGAAAGGCTTGGGTAGCAAACTTTGAAAGACCACGGATGAAT   720
18brown      TTAGAAAGACATTTAGAACGAAAGGCTTGGGTAGCAAACTTTGAAAGACCACGGATGAAT   720
27brown      TTAGAAAGACATTTAGAACGAAAGGCTTGGGTAGCAAACTTTGAAAGACCACGGATGAAT   720
20pink       TTAGAAAGACATTTAGAACGAAAGGCTTGGGTAGCAAACTTTGAAAGACCACGGATGAAT   720
30pink       TTAGAAAGACATTTAGAACGAAAGGCTTGGGTAGCAAACTTTGAAAGACCACGGATGAAT   719
Consensus    TTAGAAAGACATTTAGAACGAAAGGCTTGGGTAGCAAACTTTGAAAGACCACGGATGAAT   720
             ****************************  ******************************
```

```
AY585716      gcaaattcccttctggcaagccctacggggcttagtccttacctccgcttcggctgtctg     780
87yellow      GCAAATTCCCTTCTGGCAAGCCCTACGGGGCTTAGTCCTTACCTCCGCTTCGGCTGTCTG     780
55yellow      GCAAATTCCCTTCTGGCAAGCCCTACGGGGCTTAGTCCTTACCTCCGCTTCGGCTGTCTG     780
16white       GCAAATTCCCTTCTGGCAAGCCCTACGGGGCTTAGTCCTTACCTCCGCTTCGGCTGTCTG     780
3brown        GCAAATTCCCTTCTGGCAAGCCCTACGGGGCTTAGTCCTTACCTCCGCTTCGGCTGTCTG     780
18brown       GCAAATTCCCTTCTGGCAAGCCCTACGGGGCTTAGTCCTTACCTCCGCTTCGGCTGTCTG     780
27brown       GCAAATTCCCTTCTGGCAAGCCCTACGGGGCTTAGTCCTTACCTCCGCTTCGGCTGTCTG     780
20pink        GCAAATTCCCTTCTGGCAAGCCCTACGGGGCTTAGTCCTTACCTCCGCTTCGGCTGTCTG     780
30pink        GCAAATTCCCTTCTGGCAAGCCCTACGGGGCTTAGTCCTTACCTCCGCTTCGGCTGTCTG     779
Consensus     GCAAATTCCCTTCTGGCAAGCCCTACGGGGCTTAGTCCTTACCTCCGCTTCGGCTGTCTG     780
              ************************************************************

AY585716      tcctgccggctcttttatttcaagttaacggatctgtacaaaaaggtaaaaaagaacagc     840
87yellow      TCCTGCCGGCTCTTTTATTTCAAGTTAACGGATCTGTACAAAAAGGTAAAAAAGAACAGC     840
55yellow      TCCTGCCGGCTCTTTTATTTCAAGTTAACGGATCTGTACAAAAAGGTAAAAAAGAACAGC     840
16white       TCCTGCCGGCTCTTTTATTTCAAGTTAACGGATCTGTACAAAAAGGTAAAAAAGAACAGC     840
3brown        TCCTGCCGGCTCTTTTATTTCAAGTTAACGGATCTGTACAAAAAGGTAAAAAAGAACAGC     840
18brown       TCCTGCCGGCTCTTTTATTTCAAGTTAACGGATCTGTACAAAAAGGTAAAAAAGAACAGC     840
27brown       TCCTGCCGGCTCTTTTATTTCAAGTTAACGGATCTGTACAAAAAGGTAAAAAAGAACAGC     840
20pink        TCCTGCCGGCTCTTTTATTTCAAGTTAACGGATCTGTACAAAAAGGTAAAAAAGAACAGC     840
30pink        TCCTGCCGGCTCTTTTATTTCAAGTTAACGGATCTGTACAAAAAGGTAAAAAAGAACAGC     839
Consensus     TCCTGCCGGCTCTTTTATTTCAAGTTAACGGATCTGTACAAAAAGGTAAAAAAGAACAGC     840
              ************************************************************

AY585716      tcccctcccctctccctctatggccagctgttatggcgtgaatttttctacacagcggcg     900
87yellow      TCCCCTCCCCTCTCCCTCTATGGCCAGCTGTTATGGCGTGAATTTTTCTACACAGCGGCA     900
55yellow      TCCCCTCCCCTCTCCCTCTATGGCCAGCTGTTATGGCGTGAATTTTTCTGCACAGCGGCA     900
16white       TCCCCTCCCCTCTCCCTCTATGGCCAGCTGTTATGGCGTGAATTTTTCTACACAGCGGCA     900
3brown        TCCCCTCCCCTCTCCCTCTATGGCCAGCTGTTATGGCGTGAATTTTTCTACACAGCGGCA     900
18brown       TCCCCTCCCCTCTCCCTCTATGGCCAGCTGTTATGGCGTGAATTTTTCTACACAGCGGCA     900
27brown       TCCCCTCCCCTCTCCCTCTATGGCCAGCTGTTATGGCGTGAATTTTTCTACACAGCGGCA     900
20pink        TCCCCTCCCCTCTCCCTCTATGGCCAGCTGTTATGGCGTGAATTTTTCTACACAGCGGCA     900
30pink        TCCCCTCCCCTCTCCCTCTATGGCCAGCTGTTATGGCGTGAATTTTTCTACACAGCGGCA     899
Consensus     TCCCCTCCCCTCTCCCTCTATGGCCAGCTGTTATGGCGTGAATTTTTCTACACAGCGGCA     900
              ************************************************* *********

AY585716      actaacaatccgcggtttgataaaatggaggggaatcctatctgtgttcaaatcccatgg     960
87yellow      ACTAACAATCCGCGGTTTGATAAAATGGAGGGGAATCCTATCTGTGTTCAAATCCCATGG     960
55yellow      ACTAACAATCCGCGGTTTGATAAAATGGAGGGGAATCCTATCTGTGTTCAAATCCCATGG     960
16white       ACTAACAATCCGCGGTTTGATAAAATGGAGGGGAATCCTATCTGTGTTCAAATCCCATGG     960
3brown        ACTAACAATCCGCGGTTTGATAAAATGGAGGGGAATCCTATCTGTGTTCAAATCCCATGG     960
18brown       ACTAACAATCCGCGGTTTGATAAAATGGAGGGGAATCCTATCTGTGTTCAAATCCCATGG     960
27brown       ACTAACAATCCGCGGTTTGATAAAATGGAGGGGAATCCTATCTGTGTTCAAATCCCATGG     960
20pink        ACTAACAATCCGCGGTTTGATAAAATGGAGGGGAATCCTATCTGTGTTCAAATCCCATGG     960
30pink        ACTAACAATCCGCGGTTTGATAAAATGGAGGGGAATCCTATCTGTGTTCAAATCCCATGG     959
Consensus     ACTAACAATCCGCGGTTTGATAAAATGGAGGGGAATCCTATCTGTGTTCAAATCCCATGG     960
              ************************************************************

AY585716      gataagaatcctgaggctttggccaaatgggcagaaggcaggacaggttttccttggatt     1020
87yellow      GATAAGAATCCTGAGGCTTTGGCCAAATGGGCAGAAGGCAGGACAGGTTTTCCTTGGATT     1020
55yellow      GATAAGAATCCTGAGGCTTTGGCCAAATGGGCAGAAGGCAGGACAGGTTTTCCTTGGATT     1020
16white       GATAAGAATCCTGAGGCTTTGGCCAAATGGGCAGAAGGCAGGACAGGTTTTCCTTGGATT     1020
3brown        GATAAGAATCCTGAGGCTTTGGCCAAATGGGCAGAAGGCAGGACAGGTTTTCCTTGGATT     1020
18brown       GATAAGAATCCTGAGGCTTTGGCCAAATGGGCAGAAGGCAGGACAGGTTTTCCTTGGATT     1020
27brown       GATAAGAATCCTGAGGCTTTGGCCAAATGGGCAGAAGGCAGGACAGGTTTTCCTTGGATT     1020
20pink        GATAAGAATCCTGAGGCTTTGGCCAAATGGGCAGAAGGCAGGACAGGTTTTCCTTGGATT     1020
30pink        GATAAGAATCCTGAGGCTTTGGCCAAATGGGCAGAAGGCAGGACAGGTTTTCCTTGGATT     1019
Consensus     GATAAGAATCCTGAGGCTTTGGCCAAATGGGCAGAAGGCAGGACAGGTTTTCCTTGGATT     1020
              ************************************************************

AY585716      gatgcaattatgacacaacttcgtcaggaaggttggattcaccatttagcccggcatgct     1080
87yellow      GATGCAATTATGACACAACTTCGTCAGGAAGGTTGGATTCACCATTTAGCCCGGCATGCT     1080
55yellow      GATGCAATTATGACACAACTTCGTCAGGAAGGTTGGATTCACCATTTAGCCCGGCATGCT     1080
16white       GATGCAATTATGGCACAACTTCGTCAGGAAGGTTGGATTCACCATTTAGCCCGGCATGCT     1080
3brown        GATGCAATTATGACACAACTTCGTCAGGAAGGTTGGATTCACCATTTAGCCCGGCATGCT     1080
18brown       GATGCAATTATGACACAACTTCGTCAGGAAGGTTGGATTCACCATTTAGCCCGGCATGCT     1080
27brown       GATGCAATTATGACACAACTTCGTCAGGAAGGTTGGATTCACCATTTAGCCCGGCATGCT     1080
20pink        GATGCAATTATGACACAACTTCGTCAGGAAGGTTGGATTCACCATTTAGCCCGGCATGCT     1080
30pink        GATGCAATTATGACACAACTTCGTCAGGAAGGTTGGATTCACCATTTAGCCCGGCATGCT     1079
Consensus     GATGCAATTATGACACAACTTCGTCAGGAAGGTTGGATTCACCATTTAGCCCGGCATGCT     1080
              ************ ***********************************************
```

```
AY585716      gtcgcatgctttttgactcgaggtgacctctggattagctgggaagaaggaatgaaggtc     1140
87yellow      GTCGCATGCTTTTTGACTCGAGGTGACCTCTGGATTAGCTGGGAAGAAGGAATGAAGGTC     1140
55yellow      GTCGCATGCTTTTTGACTCGAGGTGACCTCTGGATTAGCTGGGAAGAAGGAATGAAGGTC     1140
16white       GTCGCATGCTTTTTGACTCGAGGTGACCTCTGGATTAGCTGGGAAGAAGGAATGAAGGTC     1140
3brown        GTCGCATGCTTTTTGACTCGAGGTGACCTCTGGATTAGCTGGGAAGAAGGAATGAAGGTC     1140
18brown       GTCGCATGCTTTTTGACTCGAGGTGACCTCTGGATTAGCTGGGAAGAAGGAATGAAGGTC     1140
27brown       GTCGCATGCTTTTTGACTCGAGGTGACCTCTGGATTAGCTGGGAAGAAGGAATGAAGGTC     1140
20pink        GTCGCATGCTTTTTGACTCGAGGTGACCTCTGGATTAGCTGGGAAGAAGGAATGAAGGTC     1140
30pink        GTCGCATGCTTTTTGACTCGAGGTGACCTCTGGATTAGCTGGGAAGAAGGAATGAAGGTC     1139
Consensus     GTCGCATGCTTTTTGACTCGAGGTGACCTCTGGATTAGCTGGGAAGAAGGAATGAAGGTC     1140
              ************************************************************

AY585716      tttgaagagctgttacttgatgcagattggagtgtgaatgctggaagctggatgtggctg     1200
87yellow      TTTGAAGAGCTGTTACTTGATGCAGATTGGAGTGTGAATGCTGGAAGCTGGATGTGGCTG     1200
55yellow      TTTGAAGAGCTGTTACTTGATGCAGATTGGAGTGTGAATGCTGGAAGCTGGATGTGGCTG     1200
16white       TTTGAAGAGCTGTTACTTGATGCAGATTGGAGTGTGAATGCTGGAAGCTGGATGTGGCTG     1200
3brown        TTTGAAGAGCTGTTACTTGATGCAGATTGGAGTGTGAATGCTGGAAGCTGGATGTGGCTG     1200
18brown       TTTGAAGAGCTGTTACTTGATGCAGATTGGAGTGTGAATGCTGGAAGCTGGATGTGGCTG     1200
27brown       TTTGAAGAGCTGTTACTTGATGCAGATTGGAGTGTGAATGCTGGAAGCTGGATGTGGCTG     1200
20pink        TTTGAAGAGCTGTTACTTGATGCAGATTGGAGTGTGAATGCTGGAAGCTGGATGTGGCTG     1200
30pink        TTTGAAGAGCTGTTACTTGATGCAGATTGGAGTGTGAATGCTGGAAGCTGGATGTGGCTG     1199
Consensus     TTTGAAGAGCTGTTACTTGATGCAGATTGGAGTGTGAATGCTGGAAGCTGGATGTGGCTG     1200
              ************************************************************

AY585716      tcctgtagttccttctttcaacagtttttccactgctactgcccagtgggttttggcaga     1260
87yellow      TCCTGTAGTTCCTTCTTTCAACAGTTTTTCCACTGCTACTGCCCAGTGGGTTTTGGCAGA     1260
55yellow      TCCTGTAGTTCCTTCTTTCAACAGTTTTTCCACTGCTACTGCCCAGTGGGTTTTGGCAGA     1260
16white       TCCTGTAGTTCCTTCTTTCAACAGTTTTTCCACTGCTACTGCCCAGTGGGTTTTGGCAGA     1260
3brown        TCCTGTAGTTCCTTCTTTCAACAGTTTTTCCACTGCTACTGCCCAGTGGGTTTTGGCAGA     1260
18brown       TCCTGTAGTTCCTTCTTTCAACAGTTTTTCCACTGCTACTGCCCAGTGGGTTTTGGCAGA     1260
27brown       TCCTGTAGTTCCTTCTTTCAACAGTTTTTCCACTGCTACTGCCCAGTGGGTTTTGGCAGA     1260
20pink        TCCTGTAGTTCCTTCTTTCAACAGTTTTTCCACTGCTACTGCCCAGTGGGTTTTGGCAGA     1260
30pink        TCCTGTAGTTCCTTCTTTCAACAGTTTTTCCACTGCTACTGCCCAGTGGGTTTTGGCAGA     1259
Consensus     TCCTGTAGTTCCTTCTTTCAACAGTTTTTCCACTGCTACTGCCCAGTGGGTTTTGGCAGA     1260
              ************************************************************

AY585716      agaactgacccaaatggggattatatcagacggtatttgccagtactcagaggtttccct     1320
87yellow      AGAACTGACCCAAATGGGGATTATATCAGACGGTATTTGCCAGTACTCAGAGGTTTCCCT     1320
55yellow      AGAACTGACCCAAATGGGGATTATATCAGACGGTATTTGCCAGTACTCAGAGGTTTCCCT     1320
16white       AGAACTGACCCAAATGGGGATTATATCAGACGGTATTTGCCAGTACTCAGAGGTTTCCCT     1320
3brown        AGAACTGACCCAAATGGGGATTATATCAGACGGTATTTGCCAGTACTCAGAGGTTTCCCT     1320
18brown       AGAACTGACCCAAATGGGGATTATATCAGACGGTATTTGCCAGTACTCAGAGGTTTCCCT     1320
27brown       AGAACTGACCCAAATGGGGATTATATCAGACGGTATTTGCCAGTACTCAGAGGTTTCCCT     1320
20pink        AGAACTGACCCAAATGGGGATTATATCAGACGGTATTTGCCAGTACTCAGAGGTTTCCCT     1320
30pink        AGAACTGACCCAAATGGGGATTATATCAGACGGTATTTGCCAGTACTCAGAGGTCTCCCT     1319
Consensus     AGAACTGACCCAAATGGGGATTATATCAGACGGTATTTGCCAGTACTCAGAGGTTTCCCT     1320
              ****************************************************** *****

AY585716      gcaaaatacatctatgatccttggaatgccccagagagcatccagaaggctgcaaaatgt     1380
87yellow      GCAAAATACATCTATGATCCTTGGAATGCCCCAGAGAGCATCCAGAAGGCTGCAAAATGT     1380
55yellow      GCAAAATACATCTATGATCCTTGGAATGCCCCAGAGAGCATCCAGAAGGCTGCAAAATGT     1380
16white       GCAAAATACATCTATGATCCTTGGAATGCCCCAGAGAGCATCCAGAAGGCTGCAAAATGT     1380
3brown        GCAAAATACATCTATGATCCTTGGAATGCCCCAGAGAGCATCCAGAAGGCTGCAAAATGT     1380
18brown       GCAAAATACATCTACGATCCTTGGAATGCCCCAGAGAGCATCCAGAAGGCTGCAAAATGT     1380
27brown       GCAAAATACATCTATGATCCTTGGAATGCCCCAGAGAGCATCCAGAAGGCTGCAAAATGT     1380
20pink        GCAAAATACATCTATGATCCTTGGAATGCCCCAGAGAGCATCCAGAAGGCTGCAAAATGT     1380
30pink        GCAAAATACATCTATGATCCTTGGAATGCCCCAGAGAGCATCCAGAAGGCTGCAAAATGT     1379
Consensus     GCAAAATACATCTATGATCCTTGGAATGCCCCAGAGAGCATCCAGAAGGCTGCAAAATGT     1380
              ************** *********************************************

AY585716      attataggagttaattatcccaaaccaatggtaaaccatgcagaggcaagccgtctgaat     1440
87yellow      ATTATAGGAGTTAATTATCCCAAACCAATGGTAAACCATGCAGAGGCAAGCCGTCTGAAT     1440
55yellow      ATTATAGGAGTTAATTATCCCAAACCAATGGTAAACCATGCAGAGGCAAGCCGTCTGAAT     1440
16white       ATTATAGGAGTTAATTATCCCAAACCAATGGTAAACCATGCAGAGGCAAGCCGTCTGAAT     1440
3brown        ATTATAGGAGTTAATTATCTCAAACCAATGGTAAACCATGCAGAGGCAAGCCGTCTGAAT     1440
18brown       ATTATAGGAGTTAATTATCCCAAACCAATGGTAAACCATGCAGAGGCAAGCCGTCTGAAT     1440
27brown       ATTATAGGAGTTAATTATCCCAAACCAATGGTAAACCATGCAGAGGCAAGCCGTCTGAAT     1440
20pink        ATTATAGGAGTTAATTATCCCAAACCAATGGTAAACCATGCAGAGGCAAGCCGTCTGAAT     1440
30pink        ATTATAGGAGTTAATTATCCCAAACCAATGGTAAACCATGCAGAGGCAAGCCGTCTGAAT     1439
Consensus     ATTATAGGAGTTAATTATCCCAAACCAATGGTAAACCATGCAGAGGCAAGCCGTCTGAAT     1440
              ******************* ****************************************
```

```
AY585716      attgaaaggatgaaacagatctaccagcagctttcacgatacagaggactgggtcttctt      1500
87yellow      ATTGAAAGGATGAAACAGATCTACCAGCAGCTTTCACGATACAGAGGACTGGGTCTTCTT      1500
55yellow      ATTGAAAGGATGAAACAGATCTACCAGCAGCTTTCACGATACAGAGGACTGGGTCTTCTT      1500
16white       ATTGAAAGGATGAAACAGATCTACCAGCAGCTTTCACGATACAGAGGACTGGGTCTTCTT      1500
3brown        ATTGAAAGGATGAAACAGATCTACCAGCAGCTTTCACGATACAGAGGACTGGGTCTTCTT      1500
18brown       ATTGAAAGGATGAAACAGATCTACCAGCAGCTTTCACGATACAGAGGACTGGGTCTTCTT      1500
27brown       ATTGAAAGGATGAAACAGATCTACCAGCAGCTTTCACGATACAGAGGACTGGGTCTTCTT      1500
20pink        ATTGAAAGGATGAAACAGATCTACCAGCAGCTTTCACGATACAGAGGACTGGGTCTTCTT      1500
30pink        ATTGAAAGGATGAAACAGATCTACCAGCAGCTTTCACGATACAGAGGACTGGGTCTTCTT      1499
Consensus     ATTGAAAGGATGAAACAGATCTACCAGCAGCTTTCACGATACAGAGGACTGGGTCTTCTT      1500
              ************************************************************

AY585716      gcaactgtgccttctaatccaaatggaaatggaaatggtggcctaatgggctattcacca      1560
87yellow      GCAACTGTGCCTTCTAATCCAAATGGAAATGGAAATGGTGGCCTAATGGGCTATTCACCA      1560
55yellow      GCAACAGTGCCTTCTAATCCAAATGGAAATGGAAATGGTGGCCTAATGGGCTATTCACCA      1560
16white       GCAACTGTGCCTTCTAATCCAAATGGAAATGGAAATGGTGGCCTAATGGGCTATTCACCA      1560
3brown        GCAACTGTGCCTTCTAATCCAAATGGAAATGGAAATGGTGGCCTAATGGGCTATTCACCA      1560
18brown       GCAACTGTGCCTTCTAATCCAAATGGAAATGGAAATGGTGGCCTAATGGGCTATTCACCA      1560
27brown       GCAACTGTGCCTTCTAATCCAAATGGAAATGGAAATGGTGGCCTAATGGGCTATTCACCA      1560
20pink        GCAACTGTGCCTTCTAATCCAAATGGAAATGGAAATGGTGGCCTAATGGGCTATTCACCA      1560
30pink        GCAACTGTGCCTTCTAATCCAAATGGAAATGGAAATGGTGGCCTAATGGGCTATTCACCA      1559
Consensus     GCAACTGTGCCTTCTAATCCAAATGGAAATGGAAATGGTGGCCTAATGGGCTATTCACCA      1560
              ***** ******************************************************

AY585716      ggagaaagcatttctggttgtggtagtacaggaggagctcagctgggaactggtgatggt      1620
87yellow      GGAGAAAGCATTTCTGGTTGTGGTAGTACAGGAGGAGCTCAGCTGGGAACTGGTGATGGT      1620
55yellow      GGAGAAAGCATTTCTGGTTGTGGTAGTACAGGAGGAGCTCAGCTGGGAACTGGTGATGGT      1620
16white       GGAGAAAGCATTTCTGGTTGTGGTAGTACAGGAGGAGCTCAGCTGGGAACTGGTGATGGT      1620
3brown        GGAGAAAGCATTTCTGGTTGTGGTAGTACAGGAGGAGCTCAGCTGGGAACTGGTGATGGT      1620
18brown       GGAGAAAGCATTTCTGGTTGTGGTGGTACAGGAGGAGCTCAGCTGGGAACTGGTGATGGT      1620
27brown       GGAGAAAGCATTTCTGGTTGTGGTAGTACAGGAGGAGCTCAGCTGGGAACTGGTGATGGT      1620
20pink        GGAGAAAGCATTTCTGGTTGTGGTAGTACAGGAGGAGCTCAGCTGGGAACTGGTGATGGT      1620
30pink        GGAGAAAGCATTTCTGGTTGTGGTAGTACAGGAGGAGCTCAGCTGGGAACTGGTGATGGT      1619
Consensus     GGAGAAAGCATTTCTGGTTGTGGTAGTACAGGAGGAGCTCAGCTGGGAACTGGTGATGGT      1620
              ************************ ***********************************

AY585716      catactgttgttcagtcatgtaccctgggagactctcattcaggaacaagtggaattcag      1680
87yellow      CATACTGTTGTTCAGTCATGTACCCTGGGAGACTCTCATTCAGGAACAAGTGGAATTCAG      1680
55yellow      CATACTGTTGTTCAGTCATGTACCCTGGGAGACTCTCATTCAGGAACAAGTGGAATTCAG      1680
16white       CATACTGTTGTTCAGTCATGTACCCTGGGAGACTCTCATTCAGGAACAAGTGGAATTCAG      1680
3brown        CATACTGTTGTTCAGTCATGTACCCTGGGAGACTCTCATTCAGGAACAAGTGGAATTCAG      1680
18brown       CATACTGTTGTTCAGTCATGTACCCTGGGAGACTCTCATTCAGGAACAAGTGGAATTCAG      1680
27brown       CATACTGTTGTTCAGTCATGTACCCTGGGAGACTCTCATTCAGGAACAAGTGGAATTCAG      1680
20pink        CATACTGTTGTTCAGTCATGTACCCTGGGAGACTCTCATTCAGGAACAAGTGGAATTCAG      1680
30pink        CATACTGTTGTTCAGTCATGTACCCTGGGAGACTCTCATTCAGGAACAAGTGGAATTCAG      1679
Consensus     CATACTGTTGTTCAGTCATGTACCCTGGGAGACTCTCATTCAGGAACAAGTGGAATTCAG      1680
              ************************************************************

AY585716      cagcaaggttactgtcaagcaagtagtatcttacactatgctcatggagacaatcagcaa      1740
87yellow      CAGCAAGGTTACTGTCAAGCAAGTAGTATCTTACACTATGCTCATGGAGACAATCAGCAA      1740
55yellow      CAGCAAGGTTACTGTCAAGCAAGTAGTATCTTACACTATGCTCATGGAGACAATCAGCAA      1740
16white       CAGCAAGGTTACTGTCAAGCAAGTAGTATCTTACACTATGCTCATGGAGACAATCAGCAA      1740
3brown        CAGCAAGGTTACTGTCAAGCAAGTAGTATCTTACACTATGCTCATGGAGACAATCAGCAA      1740
18brown       CAGCAAGGTTACTGTCAAGCAAGTAGTATCTTACACTATGCTCATGGAGACAATCAGCAA      1740
27brown       CAGCAAGGTTACTGTCAAGCAAGTAGTATCTTACACTATGCTCATGGAGACAATCAGCAA      1740
20pink        CAGCAAGGTTACTGTCAAGCAAGTAGTATCTTACACTATGCTCATGGAGACAATCAGCAA      1740
30pink        CAGCAAGGTTACTGTCAAGCAAGTAGTATCTTACACTATGCTCATGGAGACAATCAGCAA      1739
Consensus     CAGCAAGGTTACTGTCAAGCAAGTAGTATCTTACACTATGCTCATGGAGACAATCAGCAA      1740
              ************************************************************

AY585716      tcacacttattgcaagcaggaagaacggcccttggtactggcattagtgcagggaaacgc      1800
87yellow      TCACACTTATTGCAAGCAGGAAGAACGGCCCTTGGTACTGGCATTAGTGCAGGGAAACGC      1800
55yellow      TCACACTTATTGCAAGCAGGAAGAACGGCCCTTGGTACTGGCATTAGTGCAGGGAAACGC      1800
16white       TCACACTTATTGCAAGCAGGAAGAACGGCCCTTGGTACTGGCATTAGTGCAGGGAAACGT      1800
3brown        TCACACTTATTGCAAGCAGGAAGAACGGCCCTTGGTACTGGCATTAGTGCAGGGAAACGC      1800
18brown       TCACACTTATTGCAAGCAGGAAGAACGGCCCTTGGTACTGGCATTAGTGCAGGGAAACGC      1800
27brown       TCACACTTATTGCAAGCAGGAAGAACGGCCCTTGGTACTGGCATTAGTGCAGGGAAACGC      1800
20pink        TCACACTTATTGCAAGCAGGAAGAACGGCCCTTGGTACTGGCATTAGTGCAGGGAAACGC      1800
30pink        TCACACTTATTGCAAGCAGGAAGAACGGCCCTTGGTACTGGCATTAGTGCAGGGAAACGC      1799
Consensus     TCACACTTATTGCAAGCAGGAAGAACGGCCCTTGGTACTGGCATTAGTGCAGGGAAACGC      1800
              *********************************************************** 
```

```
AY585716     ccaaatccagaagaagaaactcagagcgttggaccaaaagtccagcgacagagcacaaat     1860
87yellow     CCAAATCCAGAAGAAGAAACTCAGAGCGTTGGACCAAAAGTCCAGCGACAGAGCACAAAT     1860
55yellow     CCAAATCCAGAAGAAGAAACTCAGAGCGTTGGACCAAAAGTCCAGCGACAGAGCACAAAT     1860
16white      CCAAATCCAGAAGAAGAAACTCAGAGCGTTGGACCAAAAGTCCAGCGACAGAGCACAAAT     1860
3brown       CCAAATCCAGAAGAAGAAACTCAGAGCGTTGGACCAAAAGTCCAGCGACAGAGCACAAAT     1860
18brown      CCAAATCCAGAAGAAGAAACTCAGAGCGTTGGACCAAAAGTCCAGCGACAGAGCACAAAT     1860
27brown      CCAAATCCAGAAGAAGAAACTCAGAGCGTTGGACCAAAAGTCCAGCGACAGAGCACAAAT     1860
20pink       CCAAATCCAGAAGAAGAAACTCAGAGCGTTGGACCAAAAGTCCAGCGACAGAGCACAAAT     1860
30pink       CCAAATCCAGAAGAAGAAACTCAGAGCGTTGGACCAAAAGTCCAGCGACAGAGCACAAAT     1859
Consensus    CCAAATCCAGAAGAAGAAACTCAGAGCGTTGGACCAAAAGTCCAGCGACAGAGCACAAAT     1860
             ************************************************************

AY585716     taa         1863
87yellow     TAA         1863
55yellow     TAA         1863
16white      TAA         1863
3brown       TAA         1863
18brown      TAA         1863
27brown      TAA         1863
20pink       TAA         1863
30pink       TAA         1862
Consensus    TAA         1863
             ***
```

erCry1b Accession number KT380949

```
AY585717       atgggggtgaacgccgtgcactggttccgcaaggggctgcggctccacgacaacccggcg  60
87yellow       ATGGGGGTGAACGCCGTGCACTGGTTCCGCAAGGGGCTGCGGCTCCACGACAACCCGGCG  60
55yellow       ATGGGGGTGAACGCCGTGCACTGGTTCCGCAAGGGGCTGCGGCTCCACGACAACCCGGCG  60
16white        ATGGGGGTGAACGCCGTGCACTGGTTCCGCAAGGGGCTGCGGCTCCACGACAACCCGGCG  60
25pink         ATGGGGGTGAACGCCGTGCACTGGTTCCGCAAGGGGCTGCGGCTCCACGACAACCCGGCG  60
94brown        ATGGGGGTGAACGCCGTGCACTGGTTCCGCAAGGGGCTGCGGCTCCACGACAACCCGGCG  60
49black        ATGGGGGTGAACGCCGTGCACTGGTTCCGCAAGGGGCTGCGGCTCCACGACAACCCGGCG  60
Consensus      ATGGGGGTGAACGCCGTGCACTGGTTCCGCAAGGGGCTGCGGCTCCACGACAACCCGGCG  60
               ************************************************************

AY585717       ctgcgggaatgcatcaggggcgccgacacggtgcgctgcgtctacatcctggacccctgg  120
87yellow       CTGCGGGAATGCATCCAGGGCGCCGACACGGTGCGCTGCGTCTACATCCTGGACCCCTGG  120
55yellow       CTGCGGGAATGCATCCAGGGCGCCGACACGGTGCGCTGCGTCTACATCCTGGACCCCTGG  120
16white        CTGCGGGAATGCATCCAGGGCGCCGACACGGTGCGCTGCGTCTACATCCTGGACCCCTGG  120
25pink         CTGCGGGAATGCATCCAGGGCGCCGACACGGTGCGCTGCGTCTACATCCTGGACCCCTGG  120
94brown        CTGCGGGAATGCATCCAGGGCGCCGACACGGTGCGCTGCGTCTACATCCTGGACCCCTGG  120
49black        CTGCGGGAATGCATCCAGGGCGCCGACACGGTGCGCTGCGTCTACATCCTGGACCCCTGG  120
Consensus      CTGCGGGAATGCATCCAGGGCGCCGACACGGTGCGCTGCGTCTACATCCTGGACCCCTGG  120
               ***************  *******************************************

AY585717       ttcgccggctcctccaacgtgggcatcaacaggtggcgattcctgcttcagtgtcttgaa  180
87yellow       TTCGCCGGCTCCTCCAACGTGGGCATCAACAGGTGGCGATTCCTGCTTCAGTGTCTTGAA  180
55yellow       TTCGCCGGCTCCTCCAACGTGGGCATCAACAGGTGGCGATTCCTGCTTCAGTGTCTTGAA  180
16white        TTCGCCGGCTCCTCCAACGTGGGCATCAACAGGTGGCGATTCCTGCTTCAGTGTCTCGAA  180
25pink         TTCGCCGGCTCCTCCAACGTGGGCATCAACAGGTGGCGATTCCTGCTTCAGTGTCTTGAA  180
94brown        TTCGCCGGCTCCTCCAACGTGGGCATCAACAGGTGGCGATTCCTGCTTCAGTGTCTCGAA  180
49black        TTCGCCGGCTCCTCCAACGTGGGCATCAACAGGTGGCGATTCCTGCTTCAGTGTCTTGAA  180
Consensus      TTCGCCGGCTCCTCCAACGTGGGCATCAACAGGTGGCGATTCCTGCTTCAGTGTCTTGAA  180
               ******************************************************** ***

AY585717       gatcttgatgccaatctacggaaactgaactcacgcttgtttgttatccgtggacagcca  240
87yellow       GATCTTGATGCCAATCTACGGAAACTGAATTCACGCTTGTTTGTTATCCGTGGACAGCCA  240
55yellow       GATCTTGATGCCAATCTACGGAGACTGAATTCACGCTTGTTTGTTATCCGTGGGCAGCCA  240
16white        GATCTTGATGCCAATCTACGGAAACTGAATTCACGCTTGTTTGTTATCCGTGGACAGCCA  240
25pink         GATCTTGATGCCAATCTACGGAAACTGAATTCACGCTTGTTTGTTATCCGTGGACAGCCA  240
94brown        GATCTTGATGCCAATCTACGGAAACTGAATTCACGCTTGTTTGTTATCCGTGGACAGCCA  240
49black        GATCTTGATGCCAATCTACGGAAACTGAATTCACGCTTGTTTGTTATCCGTGGACAGCCA  240
Consensus      GATCTTGATGCCAATCTACGGAAACTGAATTCACGCTTGTTTGTTATCCGTGGACAGCCA  240
               ********************** ****** *********************** ******
```

```
AY585717     gcagatgttttccccaggctttttaaggaatggaacattgcaaagctttctattgaatat 300
87yellow     GCAGATGTTTTCCCCAGGCTTTTTAAGGAATGGAACATTGCAAAGCTTTCTATTGAATAT 300
55yellow     GCAGATGTTTTCCCCAGGCTTTTTAAGGAATGGAACATTGCAAAGCTTTCTATTGAATAT 300
16white      GCAGATGTTTTCCCCAGGCTTTTTAAGGAATGGAACATTGCAAAGCTTTCTATTGAATAT 300
25pink       GCAGATGTTTTCCCCAGGCTTTTTAAGGAATGGAACATTGCAAAGCTTTCTATTGAATAT 300
94brown      GCAGATGTTTTCC-CAGGCTTTTTAAGGAATGGAACATTGCAAAGCTTTCTATTGAATAT 299
49black      GCAGATGTTTTCCCCAGGCTTTTTAAGGAATGGAACATTGCAAAGCTTTCTATTGAATAT 300
Consensus    GCAGATGTTTTCCCCAGGCTTTTTAAGGAATGGAACATTGCAAAGCTTTCTATTGAATAT 300
             ************* **********************************************

AY585717     gattctgaaccatttgggaaggagagagatgcagccatcaagaagctggccagtgaagct 360
87yellow     GATTCTGAACCATTTGGGAAGGAGAGAGATGCAGCCATCAAGAAGCTGGCCAGTGAAGCT 360
55yellow     GATTCTGAACCATTTGGGAAGGAGAGAGATGCAGCCATCAAGAAGCTGGCCAGTGAAGCT 360
16white      GATTCTGAACCATTTGGGAAGGAGAGAGATGCAGCCATCAAGAAGCTGGCCAGTGAAGCT 360
25pink       GATTCTGAACCATTTGGGAAGGAGAGAGATGCAGCCATCAAGAAGCTGGCCAGTGAAGCT 360
94brown      GATTCTGAACCATTTGGGAAGGAGAGAGATGCAGCCATCAAGAAGCTGGCCAGTGAAGCT 359
49black      GATTCTGAACCATTTGGGAAGGAGAGAGATGCAGCCATCAAGAAGCTGGCCAGTGAAGCT 360
Consensus    GATTCTGAACCATTTGGGAAGGAGAGAGATGCAGCCATCAAGAAGCTGGCCAGTGAAGCT 360
             ************************************************************

AY585717     ggagtggaggtcattgttcggatttctcatacattgtatgacctagacaaaatcatagaa 420
87yellow     GGAGTGGAGGTCATTGTTCGGATTTCTCATACATTGTATGACCTAGACAAAATCATAGAA 420
55yellow     GGAGTGGAGGTCATTGTTCGGATTTCTCATACATTGTATGACCTTGACAAAATCATAGAA 420
16white      GGAGTGGAGGTCATTGTTCGGATTTCTCATACATTGTATGACCTAGACAAAATCATAGAA 420
25pink       GGAGTGGAGGTCATTGTTCGGATTTCTCACACATTGTATGACCTAGACAAAATCATAGAA 420
94brown      GGAGTGGAGGTCATTGTTCGGATTTCTCATACATTGTATGACCTAGACAAAATCATAGAA 419
49black      GGAGTGGAGGTCATTGTTCGGATTTCTCATACATTGTATGACCTAGACAAAATCATAGAA 420
Consensus    GGAGTGGAGGTCATTGTTCGGATTTCTCATACATTGTATGACCTAGACAAAATCATAGAA 420
             ***************************** ************* ****************

AY585717     ttaaatgggggacagcctcctcttacttacaagcgattccagaccctaattagtagaatg 480
87yellow     TTAAATGGGGGACAGCCTCCTCTTACTTACAAGCGATTTCAGACCCTAATTAGTAGAATG 480
55yellow     TTAAATGGGGGACAGCCTCCTCTTACTTACAAGCGATTTCAGACCCTAATTAGTAGAATG 480
16white      TTAAATGGGGGACAGCCTCCTCTTACTTACAAGCGATTTCAGACCCTAATTAGTAGAATG 480
25pink       TTAAATGGGGGACAGCCTCCTCTTACTTACAAGCGATTTCAGACCCTAATTAGTAGAATG 480
94brown      TTAAATGGGGGACAGCCTCCTCTTACTTACAAGCGATTTCAGACCCTAATTAGTAGAATG 479
49black      TTAAATGGGGGACAGCCTCCTCTTACTTACAAGCGATTTCAGACCCTAATTAGTAGAATG 480
Consensus    TTAAATGGGGGACAGCCTCCTCTTACTTACAAGCGATTTCAGACCCTAATTAGTAGAATG 480
             ************************************** *********************

AY585717     gaacccctggagatgcctgtggagactataaccccagaagtaatgaagaaatgtactact 540
87yellow     GAACCCCTGGAGATGCCTGTGGAGACTATAACCCCAGAAGTAATGAAAAAATGTACTACT 540
55yellow     GAACCCCTGGAGATGCCTGTGGAGACTATAACCCCAGAAGTAATGAAAAAATGTACTACT 540
16white      GAACCCCTGGAGATGCCTGTGGAGACTATAACCCCAGAAGTAATGAAAAAATGTACTACT 540
25pink       GAACCCCTGGAGATGCCTGTGGAGACTATAACCCCAGAAGTAATGAAAAAATGTACTACT 540
94brown      GAACCCCTGGAGATGCCTGTGGAGACTATAACCCCAGAAGTAATGAAAAAATGTACTACT 539
49black      GAACCCCTGGAGATGCCTGTGGAGACTATAACCCCAGAAGTAATGAAAAAATGTACTACT 540
Consensus    GAACCCCTGGAGATGCCTGTGGAGACTATAACCCCAGAAGTAATGAAAAAATGTACTACT 540
             *********************************************** ************

AY585717     ccagtcttcgatgaccatgatgagaaatacggtgtgccatcacttgaagagctgggcttt 600
87yellow     CCAGTTTCTGATGACCATGATGAGAAATACGGTGTGCCATCACTTGAAGAGCTGGGCTTT 600
55yellow     CCAGTTTCTGATGACCATGATGAGAAATACGGTGTGCCATCACTTGAAGAGCTGGGCTTT 600
16white      CCAGTTTCTGATGACCATGATGAGAAATACGGTGTGCCATCACTTGAAGAGCTGGGCTTT 600
25pink       CCAGTTTCTGATGACCATGATGAGAAATACGGTGTGCCATCACTTGAAGAGCTGGGCTTT 600
94brown      CCAGTTTCTGATGACCATGATGAGAAATACGGTGTGCCATCACTTGAAGAGCTGGGCTTT 599
49black      CCAGTTTCTGATGACCATGATGAGAAATACGGTGTGCCATCACTTGAAGAGCTGGGCTTT 600
Consensus    CCAGTTTCTGATGACCATGATGAGAAATACGGTGTGCCATCACTTGAAGAGCTGGGCTTT 600
             ***** *  ***************************************************

AY585717     gacacagatggtctgccttctgcagtatggccagggggagaaactgaagctctcacacgc 660
87yellow     GACACAGATGGTCTGCCTTCTGCAGTATGGCCAGGGGGAGAAACTGAAGCTCTCACACGC 660
55yellow     GACACAGATGGTCTGCCTTCTGCAGTATGGCCAGGGGGAGAGACTGAAGCTCTCACACGC 660
16white      GACACAGATGGTCTGCCTTCTGCAGTATGGCCAGGGGGAGAAACTGAAGCCCTCACACGC 660
25pink       GACACAGATGGTCTGCCTTCTGCAGTATGGCCAGGGGGAGAAACTGAAGCTCTCACACGC 660
94brown      GACACAGATGGTCTGCCTTCTGCAGTATGGCCAGGGGGAGAAACTGAAGCCCTCACACGC 659
49black      GACACAGATGGTCTGCCTTCTGCAGTATGGCCAGGGGGAGAAACTGAAGCTCTCACACGC 660
Consensus    GACACAGATGGTCTGCCTTCTGCAGTATGGCCAGGGGGAGAAACTGAAGCTCTCACACGC 660
             **************************************** ********* *********
```

```
AY585717        ttagaaagacatttagaacgaaaggcttcggtagcaaactttgaaagaccacggatgaat  720
87yellow        TTAGAAAGACATTTAGAACGAAAGGCTTGGGTAGCAAACTTTGAAAGACCACGGATGAAT  720
55yellow        TTAGAAAGACATTTAGAACGAAAGGCTTGGGTAGCAAACTTTGAAAGACCACGGATGAAT  720
16white         TTAGAAAGACATTTAGAACGAAAGGCTTGGGTAGCAAACTTTGAAAGACCACGGATGAAT  720
25pink          TTAGAAAGACATTTAGAACGAAAGGCTTGGGTAGCAAACTTTGAAAGACCACGGATGAAT  720
94brown         TTAGAAAGACATTTAGAACGAAAGGCTTGGGTAGCAAACTTTGAAAGACCACGGATGAAT  719
49black         TTAGAAAGACATTTAGAACGAAAGGCCTGGGTAGCAAACTTTGAAAGACCACGGATGAAT  720
Consensus       TTAGAAAGACATTTAGAACGAAAGGCTTGGGTAGCAAACTTTGAAAGACCACGGATGAAT  720
                ************************** * *******************************

AY585717        gcaaattcccttctggcaagccctacggggcttagtccttacctccgcttcggctgtctg  780
87yellow        GCAAATTCCCTTCTGGCAAGCCCTACGGGGCTTAGTCCTTACCTCCGCTTCGGCTGTCTG  780
55yellow        GCAAATTCCCTTCTGGCAAGCCCTACGGGGCTTAGTCCTTACCTCCGCTTCGGCTGTCTG  780
16white         GCAAATTCCCTTCTGGCAAGCCCTACGGGGCTTAGTCCTTACCTCCGCTTCGGCTGTCTG  780
25pink          GCAAATTCCCTTCTGGCAAGCCCTACGGGGCTTAGTCCTTACCTCCGCTTCGGCTGTCTG  780
94brown         GCAAATTCCCTTCTGGCAAGCCCTACGGGGCTTAGTCCTTACCTCCGCTTCGGCTGTCTG  779
49black         GCAAATTCCCTTCTGGCAAGCCCTACGGGGCTTAGTCCTTACCTCCGCTTCGGCTGTCTG  780
Consensus       GCAAATTCCCTTCTGGCAAGCCCTACGGGGCTTAGTCCTTACCTCCGCTTCGGCTGTCTG  780
                ************************************************************

AY585717        tcctgccggctcttttatttcaagttaacggatctgtacaaaaaggtaaaaaagaacagc  840
87yellow        TCCTGCCGGCTCTTTTATTTCAAGTTAACGGATCTGTACAAAAAGGTAAAAAAGAACAGC  840
55yellow        TCCTGCCGGCTCTTTTATTTCAAGTTAACGGATCTGTACAAAAAGGTAAAAAAGAACAGC  840
16white         TCCTGCCGGCTCTTTTATTTCAAGTTAACGGATCTGTACAAAAAGGTAAAAAAGAACAGC  840
25pink          TCCTGCCGGCTCTTTTATTTCAAGTTAACGGATCTGTACAAAAAGGTAAAAAAGAACAGC  840
94brown         TCCTGCCGGCTCTTTTATTTCAAGTTAACGGATCTGTACAAAAAGGTAAAAAAGAACAGC  839
49black         TCCTGCCGGCTCTTTTATTTCAAGCTAACGGATCTGTACAAAAAGGTAAAAAAGAACAGC  840
Consensus       TCCTGCCGGCTCTTTTATTTCAAGTTAACGGATCTGTACAAAAAGGTAAAAAAGAACAGC  840
                ************************ ***********************************

AY585717        tcccctcccctctccctctatggccagctgttatggcgtgaatttttctacacagcggcg  900
87yellow        TCCCCTCCCCTCTCCCTCTATGGCCAGCTGTTATGGCGTGAATTTTTCTACACAGCGGCA  900
55yellow        TCCCCTCCCCTCTCCCTCTATGGCCAGCTGTTATGGCGTGAATTTTTCTACACAGCGGCA  900
16white         TCCCCTCCCCTCTCCCTCTATGGCCAGCTGTTATGGCGTGAATTTTTCTACACAGCGGCA  900
25pink          TCCCCTCCCCTCTCCCTCTATGGCCAGCTGTTATGGCGTGAATTTTTCTACACAGCGGCA  900
94brown         TCCCCTCCCCTCTCCCTCTATGGCCAGCTGTTATGGCGTGAATTTTTCTACACAGCGGCA  899
49black         TCCCCTCCCCTCTCCCTCTATGGCCAGCTGTTATGGCGTGAATTTTTCTACACAGCGGCA  900
Consensus       TCCCCTCCCCTCTCCCTCTATGGCCAGCTGTTATGGCGTGAATTTTTCTACACAGCGGCA  900
                ***********************************************************

AY585717        actaacaatccgcggtttgataaaatggaggggaatcctatctgtgttcaaatcccatgg  960
87yellow        ACTAACAATCCGCGGTTTGATAAAATGGAGGGGAATCCTATCTGTGTTCAAATCCCATGG  960
55yellow        ACTAACAATCCGCGGTTTGATAAAATGGAAGGGAATCCTATCTGTGTTCAAATCCCATGG  960
16white         ACTAACAATCCGCGGTTTGATAAAATGGAGGGGAATCCTATCTGTGTTCAAATCCCATGG  960
25pink          ACTAACAATCCGCGGTTTGATAAAATGGAGGGGAATCCTATCTGTGTTCAAATCCCATGG  960
94brown         ACTAACAATCCGCGGTTTGATAAAATGGAGGGGAATCCTATCTGTGTTCAAATCCCATGG  959
49black         ACTAACAATCCGCGGTTTGATAAAATGGAGGGGAATCCTATCTGTGTTCAAATCCCATGG  960
Consensus       ACTAACAATCCGCGGTTTGATAAAATGGAGGGGAATCCTATCTGTGTTCAAATCCCATGG  960
                ***************************** ******************************

AY585717        gataagaatcctgaggctttggccaaatgggcagaaggcaggacaggttttccttggatt  1020
87yellow        GATAAGAATCCTGAGGCTTTGGCCAAATGGGCAGAAGGCAGGACAGGTTTTCCTTGGATT  1020
55yellow        GATAAGAATCCTGAGGCTTTGGCCAAATGGGCAGAAGGCAGGACAGGTTTTCCTTGGATT  1020
16white         GATAAGAATCCTGAGGCTTTGGCCAAATGGGCAGAAGGCAGGACAGGTTTTCCTTGGATT  1020
25pink          GATAAGAATCCTGAGGCTTTGGCCAAATGGGCAGAAGGCAGGACAGGTTTTCCTTGGATT  1020
94brown         GATAAGAATCCTGAGGCTTTGGCCAAATGGGCAGAAGGCAGGACAGGTTTTCCTTGGATT  1019
49black         GATAAGAATCCTGAGGCTTTGGCCAAATGGGCAGAAGGCAGGACAGGTTTTCCTTGGATT  1020
Consensus       GATAAGAATCCTGAGGCTTTGGCCAAATGGGCAGAAGGCAGGACAGGTTTTCCTTGGATT  1020
                ************************************************************

AY585717        gatgcaattatgacacaacttcgtcaggaaggttggattcaccatttagcccggcatgct  1080
87yellow        GATGCAATTATGACACAACTTCGTCAGGAAGGTTGGATTCACCATTTAGCCCGGCATGCT  1080
55yellow        GATGCAATTATGACACAACTTCGTCAGGAAGGTTGGATTCACCATTTAGCCCGGCATGCT  1080
16white         GATGCAATTATGACACAACTTCGTCAGGAAGGTTGGATTCACCATTTAGCCCGGCATGCT  1080
25pink          GATGCAATTATGACACAACTTCGTCAGGAAGGTTGGATTCACCATTTAGCCCGGCATGCT  1080
94brown         GATGCAATTATGACACAACTTCGTCAGGAAGGTTGGATTCACCATTTAGCCCGGCATGCT  1079
49black         GATGCAATTATGACACAACTTCGTCAGGAAGGTTGGATTCACCATTTAGCCCGGCATGCT  1080
Consensus       GATGCAATTATGACACAACTTCGTCAGGAAGGTTGGATTCACCATTTAGCCCGGCATGCT  1080
                ************************************************************
```

```
AY585717        gtcgcatgctttttgactcgaggtgacctctggattagctgggaagaaggaatgaaggtc 1140
87yellow        GTCGCATGCTTTTTGACTCGAGGTGACCTCTGGATTAGCTGGGAAGAAGGAATGAAGGTC 1140
55yellow        GTCGCATGCTTTTTGACTCGAGGTGACCTCTGGATTAGCTGGGAAGAAGGAATGAAGGTC 1140
16white         GTCGCATGCTTTTTGACTCGAGGTGACCTCTGGATTAGCTGGGAAGAAGGAATGAAGGTC 1140
25pink          GTCGCATGCTTTTTGACTCGAGGTGACCTCTGGATTAGCTGGGAAGAAGGAATGAAGGTC 1140
94brown         GTCGCATGCTTTTTGACTCGAGGTGACCTCTGGATTAGCTGGGAAGAAGGAATGAAGGTC 1139
49black         GTCGCATGCTTTTTGACTCGAGGTGACCTCTGGATTAGCTGGGAAGAAGGAATGAAGGTC 1140
Consensus       GTCGCATGCTTTTTGACTCGAGGTGACCTCTGGATTAGCTGGGAAGAAGGAATGAAGGTC 1140
                ************************************************************

AY585717        tttgaagagctgttacttgatgcagattggagtgtgaatgctggaagctggatgtggctg 1200
87yellow        TTTGAAGAGCTGTTACTTGATGCAGATTGGAGTGTGAATGCTGGAAGCTGGATGTGGCTG 1200
55yellow        TTTGAAGAGCTGTTACTTGATGCAGATTGGAGTGTGAATGCTGGAAGCTGGATGTGGCTG 1200
16white         TTTGAAGAGCTGTTACTTGATGCAGATTGGAGTGTGAATGCTGGAAGCTGGATGTGGCTG 1200
25pink          TTTGAAGAGCTGTTACTTGATGCAGATTGGAGTGTGAATGCTGGAAGCTGGATGTGGCTG 1200
94brown         TTTGAAGAGCTGTTACTTGATGCAGATTGGAGTGTGAATGCTGGAAGCTGGATGTGGCTG 1199
49black         TTTGAAGAGCTGTTACTTGATGCAGATTGGAGTGTGAATGCTGGAAGCTGGATGTGGCTG 1200
Consensus       TTTGAAGAGCTGTTACTTGATGCAGATTGGAGTGTGAATGCTGGAAGCTGGATGTGGCTG 1200
                ************************************************************

AY585717        tcctgtagttccttctttcaacagtttttccactgctactgcccagtgggttttggcaga 1260
87yellow        TCCTGTAGTTCCTTCTTTCAACAGTTTTTCCACTGCTACTGCCCAGTGGGTTTTGGCAGA 1260
55yellow        TCCTGTAGTTCCTTCTTTCAACAGTTTTTCCACTGCTACTGCCCAGTGGGTTTTGGCAGA 1260
16white         TCCTGTAGTTCCTTCTTTCAACAGTTTTTCCACTGCTACTGCCCAGTGGGTTTTGGCAGA 1260
25pink          TCCTGTAGTTCCTTCTTTCAACAGTTTTTCCACTGCTACTGCCCAGTGGGTTTTGGCAGA 1260
94brown         TCCTGTAGTTCCTTCTTTCAACAGTTTTTCCACTGCTACTGCCCAGTGGGTTTTGGCAGA 1259
49black         TCCTGTAGTTCCTTCTTTCAACAGTTTTTCCACTGCTACTGCCCAGTGGGTTTTGGCAGA 1260
Consensus       TCCTGTAGTTCCTTCTTTCAACAGTTTTTCCACTGCTACTGCCCAGTGGGTTTTGGCAGA 1260
                ************************************************************

AY585717        agaactgacccaaatggggattatatcagacggtatttgccagtactcagaggtttccct 1320
87yellow        AGAACTGACCCAAATGGGGATTATATCAGACGGTATTTGCCAGTACTCAGAGGTTTCCCT 1320
55yellow        AGAACTGACCCAAATGGGGATTATATCAGACGGTATTTGCCAGTGCTCAGAGGTTTCCCT 1320
16white         AGAACTGACCCAAATGGGGATTATATCAGACGGTATTTGCCAGTACTCAGAGGTTTCCCT 1320
25pink          AGAACTGACCCAAATGGGGATTATATCAGACGGTATTTGCCAGTACTCAGAGGTTTCCCT 1320
94brown         AGAACTGACCCAAATGGGGATTATATCAGACGGTATTTGCCAGTACTCAGAGGTTTCCCT 1319
49black         AGAACTGACCCAAATGGGGATTATATCAGACGGTATTTGCCAGTACTCAGAGGTTTCCCT 1320
Consensus       AGAACTGACCCAAATGGGGATTATATCAGACGGTATTTGCCAGTACTCAGAGGTTTCCCT 1320
                ******************************************** ***************

AY585717        gcaaaatacatctatgatccttggaatgccccagagagcatccagaaggctgcaaaatgt 1380
87yellow        GCAAAATACATCTATGATCCTTGGAATGCCCCAGAGAGCATCCAGAAGGCTGCAAAATGT 1380
55yellow        GCAAAATACATCTATGATCCTTGGAATGCCCCAGAGAGCATCCAGAAGGCTGCAAAATGT 1380
16white         GCAAAATACATCTATGATCCTTGGAATGCCCCAGAGAGCATCCAGAAGGCTGCAAAATGT 1380
25pink          GCAAAATACATCTATGATCCTTGGAATGCCCCAGAGAGCATCCAGAAGGCTGCAAAATGT 1380
94brown         GCAAAATACATCTATGATCCTTGGAATGCCCCAGAGAGCATCCAGAAGGCTGCAAAATGT 1379
49black         GCAAAATACATCTATGATCCTTGGAATGCCCCAGAGAGCATCCAGAAGGCTGCAAAATGT 1380
Consensus       GCAAAATACATCTATGATCCTTGGAATGCCCCAGAGAGCATCCAGAAGGCTGCAAAATGT 1380
                ************************************************************

AY585717        attataggagttaattatcccaaaccaatggtaaaccatgcagaggcaagccgtctgaat 1440
87yellow        ATTATAGGAGTTAATTATCCCAAACCAATGGTAAACCATGCAGAGGCAAGCCGTCTGAAT 1440
55yellow        ATTATAGGAGTTAATTATCCCAAACCAATGGTAAACCATGCAGAGGCAAGCCGTCTGAAT 1440
16white         ATTATAGGAGTTAATTATCCCAAACCAATGGTAAACCATGCAGAGGCAAGCCGTCTGAAT 1440
25pink          ATTATAGGAGTTAATTATCCCAAACCAATGGTAAACCATGCAGAGGCAAGCCGTCTGAAT 1440
94brown         ATTATAGGAGTTAATTATCCCAAACCAATGGTAAACCATGCAGAGGCAAGCCGTCTGAAT 1439
49black         ATTATAGGAGTTAATTATCCCAAACCAATGGTAAACCATGCAGAGGCAAGCCGTCTGAAT 1440
Consensus       ATTATAGGAGTTAATTATCCCAAACCAATGGTAAACCATGCAGAGGCAAGCCGTCTGAAT 1440
                ************************************************************

AY585717        attgaaaggatgaaacagatctaccagcagctttcacgatacagaggactgggtcttctt 1500
87yellow        ATTGAAAGGATGAAACAGATCTACCAGCAGCTTTCACGATACAGAGGACTGGGTCTTCTT 1500
55yellow        ATTGAAAGGATGAAACAGATCTACCAGCAGCTTTCACGATACAGAGGACTGGGTCTTCTT 1500
16white         ATTGAAAGGATGAAACAGATCTACCAGCAGCTTTCACGATACAGAGGACTGGGTCTTCTT 1500
25pink          ATTGAAAGGATGAAACAGATCTACCAGCAGCTTTCACGATACAGAGGACTGGGTCTTCTT 1500
94brown         ATTGAAAGGATGAAACAGATCTACCAGCAGCTTTCACGATACAGAGGACTGGGTCTTCTT 1499
49black         ATTGAAAGGATGAAACAGATCTACCAGCAGCTTTCACGATACAGAGGACTGGGTCTTCTT 1500
Consensus       ATTGAAAGGATGAAACAGATCTACCAGCAGCTTTCACGATACAGAGGACTGGGTCTTCTT 1500
                ************************************************************
```

```
AY585717      gcaactgtgccttctaatccaaatggaaatggaaatggtggcctaatgggctattcacca 1560
87yellow      GCAACTGTGCCTTCTAATCCAAATGGAAATGGAAATGGTGGCCTAATGGGCTATTCACCA 1560
55yellow      GCAACTGTGCCTTCTAATCCAAATGGAAATGGAAATGGTGGCCTAATGGGCTATTCACCA 1560
16white       GCAACTGTGCCTTCTAATCCAAATGGAAATGGAAATGGTGGCCTAATGGGCTATTCACCA 1560
25pink        GCAACTGTGCCTTCTAATCCAAATGGAAATGGAAATGGTGGCCTAATGGGCTATTCACCA 1560
94brown       GCAACTGTGCCTTCTAATCCAAATGGAAATGGAAATGGTGGCCTAATGGGCTATTCACCA 1559
49black       GCAACTGTGCCTTCTAATCCAAATGGAAATGGAAATGGTGGCCTAATGGGCTATTCACCA 1560
Consensus     GCAACTGTGCCTTCTAATCCAAATGGAAATGGAAATGGTGGCCTAATGGGCTATTCACCA 1560
              ************************************************************

AY585717      ggagaaagcatttctggttgtggtagtacaggaggagctcagctgggaactggtgatggt 1620
87yellow      GGAGAAAGCATTTCTGGTTGTGGTAGTACAGGAGGAGCTCAGCTGGGAACTGGTGATGGT 1620
55yellow      GGAGAAAGCATTTCTGGTTGTGGTAGTACAGGAGGAGCTCAGCTGGGAACTGGTGATGGT 1620
16white       GGAGAAAGCATTTCTGGTTGTGGTAGTACAGGAGGAGCTCAGCTTGGAACTGGTGATGGT 1620
25pink        GGAGAAAGCATTTCTGGTTGGGGTAGTACAGGAGGAGCTCAGCTGGGAACTGGTGATGGT 1620
94brown       GGAGAAAGCATTTCTGGTTGTGGTAGTACAGGAGGAGCTCAGCTTGGAACTGGTGATGGT 1619
49black       GGAGAAAGCATTTCTGGTTGTGGTAGTACAGGAGGAGCTCAGCTGGGAACTGGTGATGGT 1620
Consensus     GGAGAAAGCATTTCTGGTTGTGGTAGTACAGGAGGAGCTCAGCTGGGAACTGGTGATGGT 1620
              ******************** *********************** ***************

AY585717      catactgttgttcagtcatgtaccctgggagactctcattcaggaacaagtggaattcag 1680
87yellow      CATACTGTTGTTCAGTCATGTACCCTGGGAGACTCTCATTCAGGAACAAGTGGAATTCAG 1680
55yellow      CATACTGTTGTTCAGTCATGTACCCTGGGAGACTCTCATTCAGGAACAAGTGGAATTCAG 1680
16white       CATACTGTTGTTCAGTCATGTACCCTGGGAGACTCTCATTCAGGAACAAGTGGAATTCAG 1680
25pink        CATACTGTTGTTCAGTCATGTACCCTGGGAGACTCTCATTCAGGAACAAGTGGAATTCAG 1680
94brown       CATACTGTTGTTCAGTCATGTACCCTGGGAGACTCTCATTCAGGAACAAGTGGAATTCAG 1679
49black       CATACTGTTGTTCAGTCATGTACCCTGGGAGACTCTCATTCAGGAACAAGTGGAATTCAG 1680
Consensus     CATACTGTTGTTCAGTCATGTACCCTGGGAGACTCTCATTCAGGAACAAGTGGAATTCAG 1680
              ************************************************************

AY585717      cagcaaggtattatggcagtgcctgtctgtagaggatctccaaatgcttgcaactatgga 1740
87yellow      CAGCAAGGTATTATGGCAGTGCCTGTCTGTAGAGGATCTCCAAATGCTTGCAACTATGGA 1740
55yellow      CAGCAAGGTATTATGGCAGTGCCTGTCTGTAGAGGATCTCCAAATGCTTGCAACTATGGA 1740
16white       CAGCAAGGTATTATGGCAGTGCCTGTCTGTAGAGGATCTCCAAATGCTTGCAACTATGGA 1740
25pink        CAGCAAGGTATTATGGCAGTGCCTGTCTGTAGAGGATCTCCAAATGCTTGCAACTATGGA 1740
94brown       CAGCAAGGTATTATGGCAGTGCCTGTCTGTAGAGGATCTCCAAATGCTTGCAACTATGGA 1739
49black       CAGCAAGGTATTATGGCAGTGCCTGTCTGTAGAGGATCTCCAAATGCTTGCAACTATGGA 1740
Consensus     CAGCAAGGTATTATGGCAGTGCCTGTCTGTAGAGGATCTCCAAATGCTTGCAACTATGGA 1740
              ************************************************************

AY585717      aaaccagacaaaacatcaaaatag    1764
87yellow      AAACCAGACAAAACATCAAAATAG    1764
55yellow      AAACCAGACAAAACATCAAAATAG    1764
16white       AAACCAGACAAAACATCAAAATAG    1764
25pink        AAACCAGACAAAACATCAAAATAG    1764
94brown       AAACCAGACAAAACATCAAAATAG    1763
49black       AAACCAGACAAAACATCAAAATAG    1764
Consensus     AAACCAGACAAAACATCAAAATAG    1764
              ************************
```
